# Predicting chemical structure using reinforcement learning with a stack-augmented conditional variational autoencoder

**DOI:** 10.1186/s13321-022-00666-9

**Published:** 2022-12-09

**Authors:** Hwanhee Kim, Soohyun Ko, Byung Ju Kim, Sung Jin Ryu, Jaegyoon Ahn

**Affiliations:** 1 Department of Computer Science and Engineering, Incheon National University, Incheon, 22012 Republic of Korea; 2 GenesisEgo, Seoul, 04382 Republic of Korea; 3 UBLBio Corporation, Suwon, 16679 Republic of Korea

**Keywords:** De novo drug design, Reinforcement learning, Conditional Variational AutoEencoder, Sorafenib, Raf kinases

## Abstract

In this paper, a reinforcement learning model is proposed that can maximize the predicted binding affinity between a generated molecule and target proteins. The model used to generate molecules in the proposed model was the Stacked Conditional Variation AutoEncoder (Stack-CVAE), which acts as an agent in reinforcement learning so that the resulting chemical formulas have the desired chemical properties and show high binding affinity with specific target proteins. We generated 1000 chemical formulas using the chemical properties of sorafenib and the three target kinases of sorafenib. Then, we confirmed that Stack-CVAE generates more of the valid and unique chemical compounds that have the desired chemical properties and predicted binding affinity better than other generative models. More detailed analysis for 100 of the top scoring molecules show that they are novel ones not found in existing chemical databases. Moreover, they reveal significantly higher predicted binding affinity score for Raf kinases than for other kinases. Furthermore, they are highly druggable and synthesizable.

**Supplementary Information:**

The online version contains supplementary material available at 10.1186/s13321-022-00666-9.

## Background

The goal of drug discovery is to identify novel molecules with desired chemical or pharmacological properties. However, the search space for identification of such molecules is vast and discontinuous, which makes drug discovery difficult and costly [1]. To address this problem, many computational methods have been studied [2] and Computer-Aided Drug Discovery (CADD), such as Virtual Screening and Structure/Ligand-Based Drug Design, have been applied for drug discovery [3–5].

Recently, various deep generative models have been proposed and used to solve a variety of problems in drug discovery [6]. Many deep learning-based drug discovery studies express the chemical structure using the Simplified Molecular Input Line Entry System (SMILES) [7]. Models for generating SMILES expressions of chemicals include Recurrent Neural Network (RNN)-based models [8–10], Generative Adversarial Network (GAN)-based models [11], and AutoEncoder (AE)-based models [12–15] and their combinations.

The RNNs and their variations are powerful generative models, especially in natural language processing. Because SMILES is a string of characters, it is also effective for SMILES generation. However, while RNN is suitable for a training set, it may have limited ability to generate novel chemicals that are significantly different from the training set [10]. For this reason, RNNs and their variations are often used as the generators of GAN, or decoders and encoders of AE. For example, Guimaraes et al. [16] proposed a sequence-based GAN framework named objective-reinforced generative adversarial network (ORGAN). In this model, a CNN model was used as the discriminator to classify tests, and an RNN model with LSTM units was used as the generator.

GANs often give better results than other generation methods. However, a GAN has two main problems. First, a GAN is hard to converge and is unstable. For successful training of GAN-based models, a relatively large amount of computing and human effort is required. Another problem is the mode collapse problem. Generators of GAN-based models are trained to deceive the discriminator and cannot capture multimode distributions of real data [17].

Compared to GAN, an AE and its variations are relatively easy to train. For drug design, the encoder and decoder of AEs and their variations such as VAE (Variational AutoEncoder) and CVAE (Conditional Variational AutoEncoder) are often implemented using RNNs and their variations. A SMILE string can be generated by putting latent vectors made by the encoder into a decoder and using a predictor to measure the chemical properties [8]. A new molecule with the desired properties can be created by attaching conditions to the input data and latent vectors [9].

Meanwhile, reinforcement learning can be used to fine-tune pre-trained models [12–14, 18–20].In ReLeaSE [13], the policy to select a behavior is a generation model that is implemented using a stack-augmented RNN (Stack-RNN). The reward to learn policies is measured through the properties of the generated chemicals, and the generated model is retrained to have the desired chemical properties. PaccManRL [14] applied reinforcement learning to create an effective anticancer drug structure for given transcriptomic profiles. PaccManRL is trained to maximize a reward that is calculated by a drug sensitivity prediction model called PaccMann [15]. Olivecrona et al. [20] proposed a reinforcement learning model to generate drugs similar to existing drugs. This model measures the similarity between a specific drug and SMILES produced by the model and is trained to maximize the similarity. Liu et al. [18] introduced an RNN model with an exploration strategy. This model was trained with a reward that is created by a pre-trained predictor to predict whether the molecules generated are active or not.

The proposed method exploits not only the chemical properties of molecules but also the binding affinity between molecules and target proteins, that is, to where molecules should or should not bind. For a generative model, we designed Stack-CVAE, which applies Stack-RNN [19] to CVAE. We can input the desired chemical properties such as molecular weight, LogP, and the topological polar surface area (TPSA) [21] into Stack-CVAE. Then reinforcement learning is used to maximize or minimize the binding affinity calculated using DeepPurpose [22], and to maximize RAscore [23] to increase the synthesizability of the generated drugs.

In this study, we aimed to generate structures of a targeted anticancer drug of which indications and chemical properties are equivalent to or better than those of sorafenib. To achieve this goal, we first pre-trained Stack-CVAE using 1.5 million chemicals in the ChEMBL database [24] with the molecular weight, LogP, and TPSA of sorafenib. Then reinforcement learning was performed to maximize the RAscore and binding affinity score to sorafenib target Raf kinases (A-Raf, B-Raf, and C-Raf). Reinforcement learning was also used to minimize the binding affinity to sorafenib non-target kinases (ERK-1, MEK-1, EGFR, HER-2, IGFR-1, c-met, PKB, PKA, CDK1, PKCα, PKC γ, and pim-1) [25].

We generated 1000 chemical formulas and confirmed that Stack-CVAE generates more of the valid and unique chemical compounds that have the desired chemical properties and predicted binding affinity than other generative models do. For more detailed analysis, we selected 100 of the top scoring molecules from among 1000 molecules. The top 100 molecules are novel ones not found in existing chemical databases and have significantly higher binding affinity for Raf kinases. Their Druglikeness (DL), TPSA, and calculated LogP (cLogP) are similar to those of pre-existing drugs, and in silico ADME and toxicity profiles show that they are druggable. Furthermore, the synthetic accessibility (SA) score was comparable to those of approved drugs, which shows that they are synthesizable.

## Methods

### Conditional variational autoencoder with Stack augmented RNN (Stack-CVAE)

We designed a novel generative model, Stack-CVAE, that combines stack-RNN with CVAE to generate structural expressions in SMILES. Stack-CVAE is based on CVAE, which produces substances similar to, but not the same as, the substances used for training. The objective function of CVAE is as follows:

1 $$E\left[\mathrm{log}P\left(z,c\right)\right]-{D}_{KL}\left[Q\left(z|X,c\right)\Vert P\left(z|c\right)\right]$$

In formula (1), $Q(z|X,c)$ and $P(z|c)$ approximate the probability distributions of an encoder and a decoder, respectively. The term ${D}_{KL}$ is the Kullback–Leibler divergence, and $X$ and $z$ indicate input data and latent spaces, respectively. Here, the term $c$ indicates a condition vector that is associated with encoding and decoding.

The biggest difference between Stack-CVAE and CVAE is that CVAE uses regular RNN, LSTM, or GRU, and Stack-CVAE uses stack-augmented RNN (Stack-RNN). Stack-RNN is an augmented recurrent network with structured and growing memory. Stack-RNN has three operations. The *POP* operation deletes an element of a stack; the *PUSH* operation adds a new element to the top of a stack; and the *NO-OP* operation does not do anything. One of three operations is selected at each time step by a three-dimensional variable ${a}_{t}$, which is calculated using a hidden variable ${h}_{t}$ as in formula (2).

2 $${a}_{t}=f\left({Ah}_{t}\right)$$

In formula (2), $A$ is a $3\times m$ matrix ($m$ is the size of the hidden layer) and $f$ is a Softmax function. We denote ${a}_{t}\left[PUSH\right]$ by the probability of the *PUSH* operation, ${a}_{t}\left[POP\right]$ by the probability of the *POP* operation and ${a}_{t}\left[NO-OP\right]$ by the probability of the *NO-OP* operation. The stack is stored in a vector ${s}_{t}$ with size *p* at time ***t***, and *p* is not fixed in order to increase the capacity of the model. The top element is stored in position 0 with value of ${s}_{t}\left[0\right]$. The *PUSH* operation adds a new element to the position 0 as in formula (3).

3 $${s}_{t}\left[0\right]={a}_{t}\left[PUSH\right]\sigma \left({Dh}_{t}\right)+{a}_{t}\left[POP\right]{s}_{t-1}\left[1\right]+{a}_{t}\left[NO-OP\right]{s}_{t-1}\left[0\right]$$

where $D$ is $1\times m$ matrix. If ${a}_{t}\left[POP\right]$ is equal to 1, the top element is popped and all the other elements are moved up one position. If ${a}_{t}\left[PUSH\right]$ is 1, all elements are moved down one position and the new element is added to the top of the stack. If ${a}_{t}\left[NO-OP\right]$ is 1, the stack is not changed. Similarly, for an element stored at a depth > 0 in the stack, elements in the previous stack are stored in the current stack as in the formula (4).

4 $${s}_{t}\left[i\right]={a}_{t}\left[PUSH\right]{s}_{t-1}\left[i-1\right]+{a}_{t}\left[POP\right]{s}_{t-1}\left[i+1\right]\left(i>0\right)+{a}_{t}\left[NO-OP\right]{s}_{t-1}\left[i\right]$$

An element of a stack is propagated to the next hidden layer, which is calculated by formula (5).

5 $${h}_{t}=\sigma \left(U{x}_{t}+R{h}_{t-1}+P{s}_{t-1}^{k}\right)$$

**Fig. 1:**
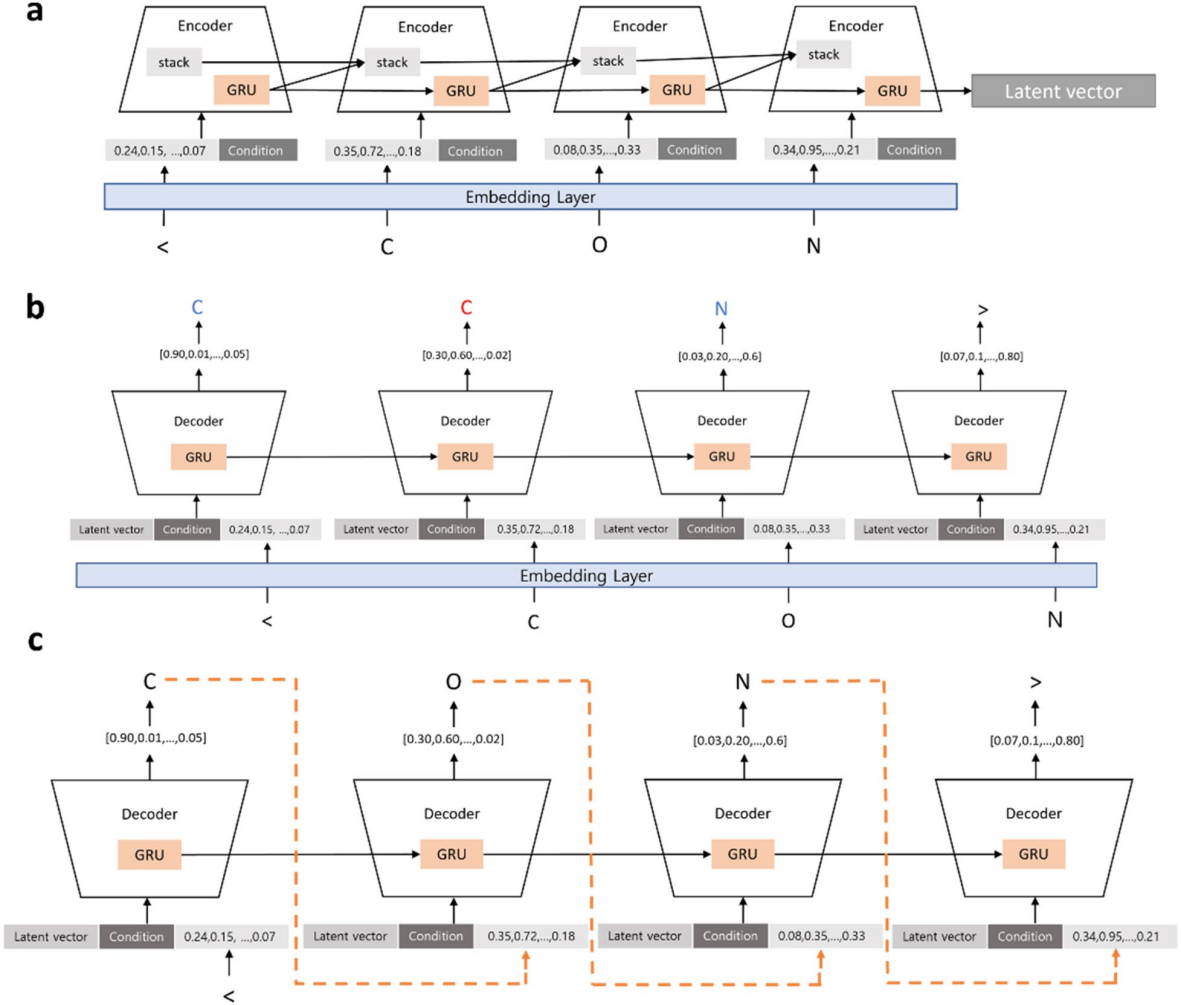
Training and generation steps: Training steps of encoder and decoder of stack-CVAE are shown in **a** and **b**, respectively. Generation of SMILES by a decoder of stack-CVAE is shown in **c**

In formula (5), $P$ is $m\times k$ matrix, ${s}_{t-1}^{k}$ is a top element of the stack of time point *t*-1, and $k$ is the size of an element. Because Stack-RNN is an RNN of which a cell has a stack structure, it has strength to learn longer and more complex data. Stack memory increases the probability of generating valid SMILES because RNN without a stack structure cannot learn ring structure or brackets. Furthermore, RNN without stack structure tends to generate SMILES that are similar to the training SMILES [16]. Because Stack-CVAE uses Stack-RNN as its encoder, the proportion of valid and unique SMILES of Stack-CVAE can be higher than that of CVAE of which the encoder is an RNN, GRU, or LSTM. We used GRU as a decoder. The model is depicted in Fig. 1.

First, the '<' character, which indicates the start of a SMILES, is put in front of the SMILES string, and the '>' character, which indicates the end of a SMILES, is put at the end of a SMILES string. When input SMILES is put into the generation model, an embedding vector is generated and combined with a pre-produced condition vector to generate an input matrix. The condition vector is made by calculating the molecular weight, LogP, and TPSA of input the SMILES using RDkit [26]. The input matrix generated is input to the encoder of the Stack-CVAE and converted into a latent vector. The process for generating a latent vector is illustrated in Fig. 1a. The encoder of Stack-CVAE used a 3-layer Stack-RNN with 512 hidden nodes, a stack-width of size 50, a stack-depth of size 10, and a 3-layer GRU with 512 hidden nodes as a decoder. The cross entropy was used as the cost function of the reconstruct error, and a linear neural network was used for each output of the decoder cell.

A generated latent vector is combined with the condition vector and input to the decoder. Finally, the output of the decoder generates a distribution of the probability that each token will be selected through Softmax and the decoder is trained to predict a token correctly. This process is illustrated in Fig. 1b.

After training, a new molecule can be generated using the generator, that is, the decoder of stack-CVAE. When the start token '<' is entered, the decoder generates a new token. A generated token is re-entered into a decoder and this process is repeated until a decoder generates an end token '>'. This process is illustrated in Fig. 1c.

### Reinforcement learning for drug properties

In the pre-training stage, the Stack-CVAE model learns the rules of SMILES given conditions such as molecular weight, LogP, and TPSA. Then, reinforcement learning is applied to the pre-trained Stack-RNN model to generate a new chemical compound with the desired properties. In reinforcement learning, an agent determines a behavior according to a policy that specifies the probability of taking an action in a state. An environment then rewards an agent, and an agent is trained based on rewards. Reinforcement learning is performed to maximize accumulated rewards returned by an environment.

In the proposed model, an agent corresponds to a decoder of stack-CVAE, and a policy is a probability distribution of a decoder of stack-CVAE to generate tokens. An action and a state of an agent mean creating one token and a generated token, respectively. When all actions are completed, a chemical formula in the form of SMILES is generated, and the reward is measured by calculating the RAscore and binding affinity of the SMILES generated.

More specifically, a state of a policy means the current token. If a current token is combined with the latent vector *z* generated using a random value from a normal distribution with *mean* = 0 and *sd* = 1, and put into a decoder of stack-CVAE, then the probability distribution for the next token given the latent vector *z* is calculated. The next token is sampled according to the probability distribution, and this process is repeated until the ending character '>' is generated. Until then, the reward is zero, and an environment calculates reward based on two predicted properties of the generated SMILES.

The first property is the predicted Retrosynthetic Accessibility score (RAscore), which is calculated by a synthesis planning tool, AiZynthFinder [27]. RAscore is a coefficient indicating the synthesis possibility and has the value 1 if synthesis is possible, and 0 if not. The reward by RAscore is *reward1* and is calculated by formula (6).

6 $$reward1= \left\{\begin{array}{cc}1, & if\;RAscore=0\\ RAscore \times 5+1, & otherwise\end{array}\right.$$

The second property is the binding affinity of the produced SMILES to the target proteins. The binding affinity was calculated using DeepPurpose. For multiple target proteins, binding affinities were averaged. We had two groups of target proteins, group A and group D. Group A included target proteins that should bind to the generated SMILES, and group D included target proteins that should not bind to the generated SMILES. The averaged binding affinity for group A and D is *affA* and *affD*, respectively. The reward by the binding affinity to group A is *reward2* and is calculated by formula (7).

7 $$reward2= \left\{\begin{array}{cc}{(affA-4)}^{2}+1, & if \; affA > 5\\ 1, & otherwise\end{array}\right.$$

Reward by binding affinity to group D is *reward3*, and is 6, if *affD* < 5.5 and 1, otherwise. The entire reward is a sum of *reward1*, *reward2*, and *reward3*.

A decoder of stack-CVAE is trained to increase the reward and decrease loss, which is calculated by formula (8).

8 $$Loss=-\sum_{t=0}^{T-1}{\log}\left(y\right)\times discount\;reward$$

**Fig. 2:**
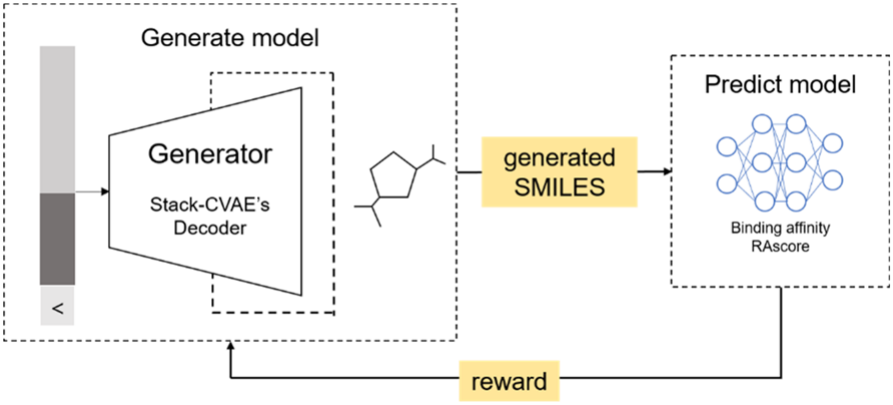
Pipeline of the reinforcement learning model for novel molecule generation

In formula (8), $T$ means the length of the generated SMILES string, and $y$ is the probability that the $(t+1)$ th token appears. Discount reward is reward multiplied by a discount rate, which we set to 0.1. The overall reinforcement learning process is illustrated in Fig. 2.

## Results and discussion

### Model performance

The Stack-CVAE model was trained with 1,499,939 SMILES data collected from the ChEMBL database. The length of the SMILES strings was limited to < 100, and molecular weight was limited to between 150 and 500. After pre-training, we generated 10,000 SMILES strings and found 6,463 SMILES strings among them that were valid.

In this study, we generated SMILES that show not only specific binding to Raf kinases, but also have properties that are equivalent to or better than those of sorafenib, a Raf kinase inhibitor. Sorafenib is a multikinase inhibitor and is known to inhibit Raf as well as other kinases [25, 28–31]. Recent studies show that sorafenib has inhibitory activity on more than 99 kinases at clinically relevant concentrations [32, 33]. This property may not only reduce the therapeutic efficacy of sorafenib, but could also cause toxicity due to increased drug use. Therefore, it is crucial to develop therapeutic drugs that can only target Raf kinases (i.e., on-target) rather than non-Raf kinases (i.e., off-target). To this end, we used a Stack-CVAE model to make Raf kinase-specific SMILES. We used three Raf kinases (A-Raf, B-Raf, and C-Raf) as sorafenib targets and ten non-Raf kinases (ERK-1, MEK-1, EGFR, HER-2, IGFR-1, c-met, PKB, PKA, CDK1, PKCα, PKCγ, and pim-1) as non-sorafenib targets.

**Fig. 3:**
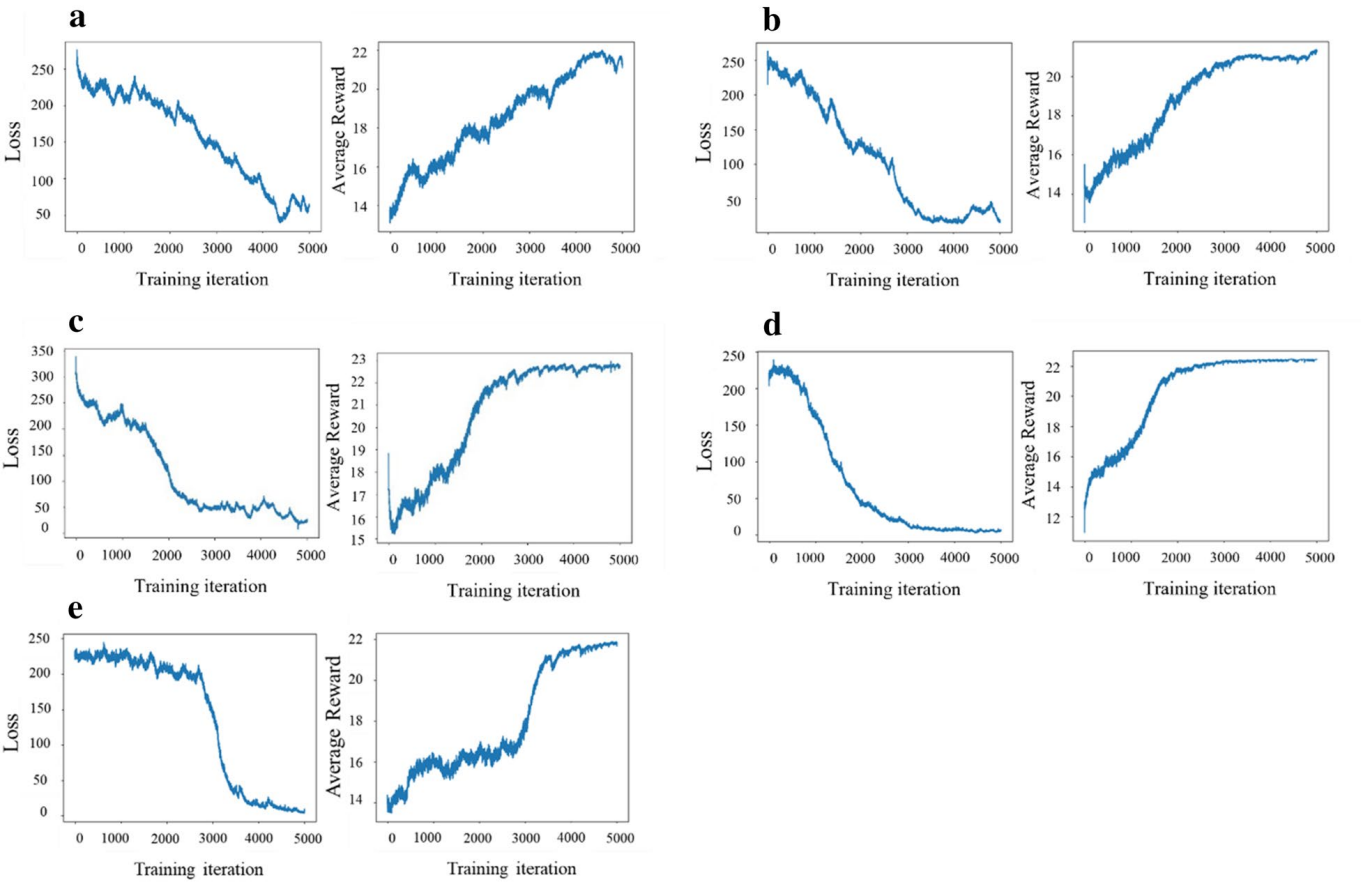
Loss and reward graphs of different generative models: Loss and Rewards graphs of stack-CVAE (**a**), CVAE (**b**), SSVAE(**c**), stack-RNN (**d**) and cRNN (**e**)

Because Stack-CVAE is based on CVAE and Stack-RNN, we performed reinforcement learning and compared results using pre-trained Stack-CVAE, CVAE, and Stack-RNN as a policy. We also used SSVAE [34] and cRNN [35] as a policy for a comparison test. Figure 3 shows that loss and reward of three generation models decreases and increases, respectively, as training epochs increase.

After reinforcement learning with 500 epochs, we generated 1000 SMILES strings for Stack-CVAE, CVAE, and Stack-RNN. The results are summarized in Table 1, which shows that, although Stack-RNN has higher probability of valid molecules, Stack-CVAE has much higher probability of valid and unique molecules than other algorithms do. Moreover, we can see that Stack-RNN generates molecules that are the same as the training molecules.

**Table 1:** Proportion of valid and unique molecules

	% of valid SMILES	% of valid and unique SMILES	% of valid and unique SMILES not in training data
Stack-CVAE (%)	98.1	81.1	81.1
CVAE (%)	98.1	39.9	39.9
SSVAE (%)	98.8	16.6	16.6
Stack-RNN (%)	100	1.5	1.2
cRNN (%)	99.7	25.2	25.2

Stack-CVAE can generate a greater number of valid and diverse molecules. However, diverse molecules would be meaningless if they do not show the desired properties. To show the proportion of molecules that show desired properties, we calculated the number of SMILES strings of which binding affinity to on-targets and off-targets, RAscore, molecular weight, logP, and TPSA values fell within 10% range of those of sorafenib (among the valid and unique SMILES not in training data for Stack-CVAE, SSVAE, cRNN, CVAE, and StackRNN). Table 2 shows the summarized results. Compared to CVAE, Stack-CVAE has higher probability of generating molecules with the desired properties, except for on-target binding affinity. SSVAE and cRNN show higher probability on RAscore, LogP, Molecular Weight and/or TPSA compared to Stack-CVAE, but the number of molecules is far less than that of Stack-CVAE.

**Table 2:** Molecules having desired properties among valid and unique SMILES not in training data

	Stack-CVAE	SSVAE	cRNN	CVAE	Stack-RNN
Binding Affinity on on-target proteins	652 (80.39%)	133 (80.12%)	145(57.54%)	368 (92.23%)	11 (91.67%)
Binding Affinity on off-target proteins	810 (99.88%)	139 (83.73%)	236(93.65%)	392 (98.25%)	12 (100%)
RAscore	566 (69.79%)	158 (95.18%)	248(98.41%)	198 (49.62%)	1 (8.33%)
Molecular Weight	532 (65.60%)	147 (88.55%)	198(78.57%)	134 (33.58%)	0 (0%)
LogP	623 (76.82%)	129 (77.71%)	180(71.43%)	238 (59.65%)	0 (0%)
TPSA	594 (73.24%)	138 (83.13%)	194(76.98%)	278 (69.67%)	0 (0%)

### Quantitative and qualitative analysis of the top 100 scoring molecules

We scored 1000 molecules according to the formula (9).

9 $$\mathrm{score}=\frac{1}{(0.3 \times \frac{mw+logP+TPSA}{3}+0.7 \times \frac{bindA+1-bindD+1-RAscore}{3})}$$

where *mw*, *logP*, and *TPSA* are 0–1 scaled differences of molecular weights, logP, and TPSA of sorafenib and generated molecules, respectively. The items *bindA* and *bindD* are 0–1 scaled values of an average binding affinity between a generated molecule and on-target and off-target proteins, respectively. Molecules with high scores are likely to have more chemical properties similar to those of sorafenib. We selected 100 of the top scoring molecules and performed more detailed analysis. The top 100 molecules are shown in Additional file 1: Fig. S1.

**Fig. 4:**
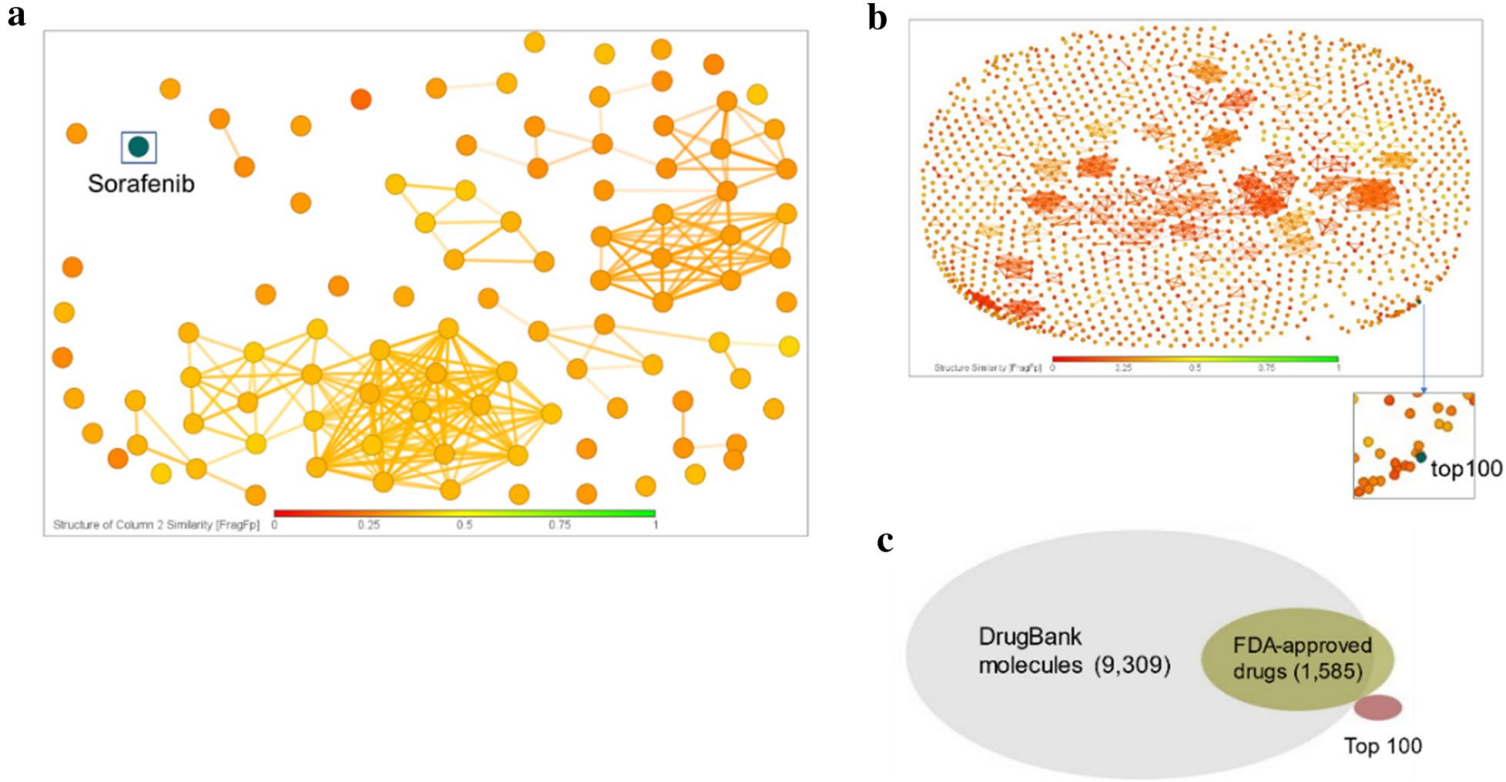
Novelty of the top 100 molecules: Structures of the top 100 scoring molecules are not similar to that of sorafenib (**a**) and FDA-approved drugs (**b**), within a similarity limit of 95%. The top 100 scoring molecules do not overlap DrugBank and FDA-approved drugs within a similarity limit of 90% (see **c**). All analyses were performed using *DataWarrior* 5.5.0

First, we checked the novelty of the top 100 molecules. Figure 4a and b show that the top 100 molecules are dissimilar to sorafenib and 1,585 FDA-approved drugs, respectively. We also checked that the top 100 molecules did not overlap with 9,309 DrugBank [36] drugs and 1,585 FDA-approved drugs within the similarity limit of 90%. All drug data were downloaded from *DataWarrior* 5.5.0 [37].

**Fig. 5:**
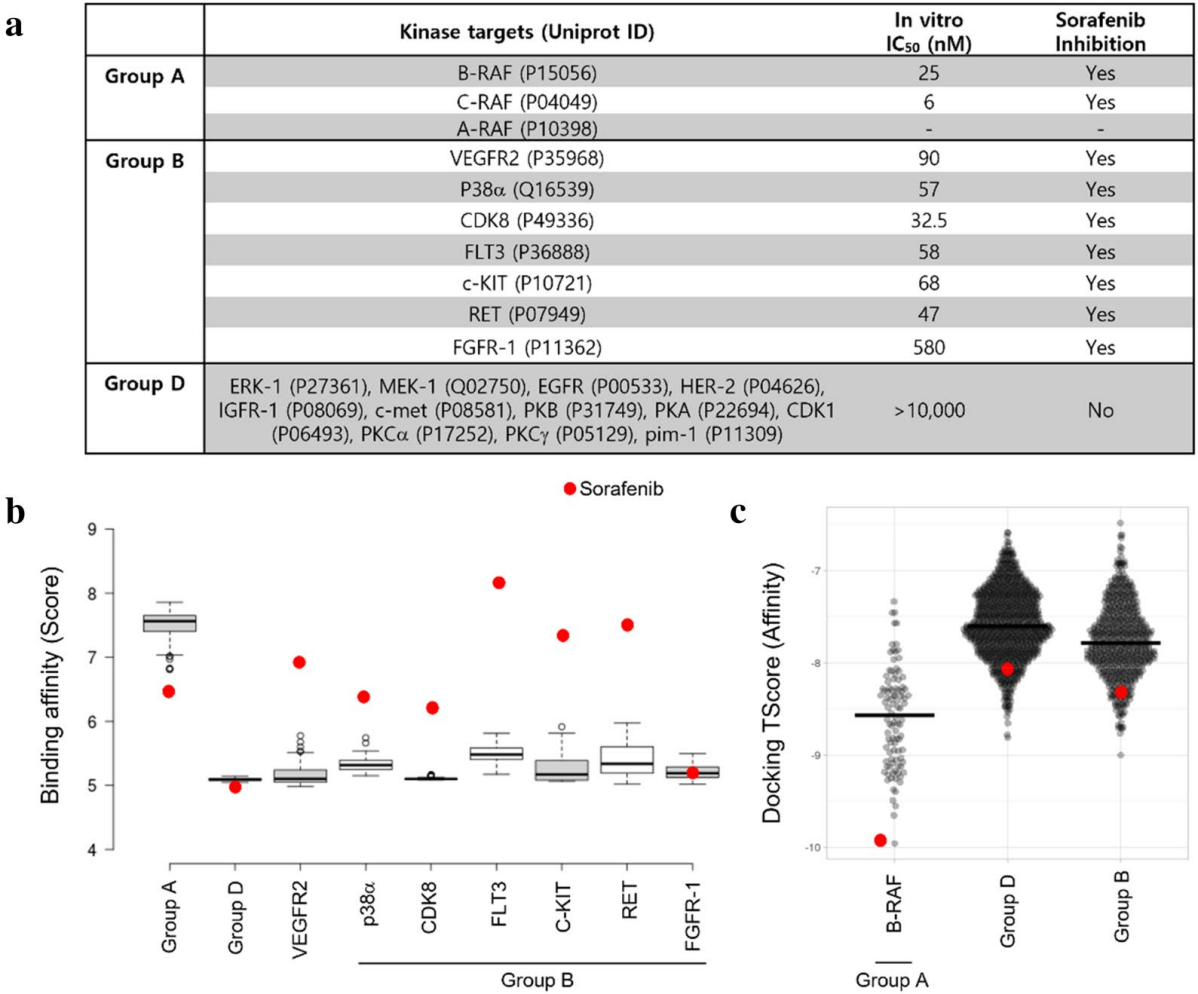
Binding specificity of the top 100 molecules: List of kinase proteins used for study is shown in **a**. Binding of the top 100 molecules is specific to Raf kinases, but not to other kinases (see **b**). Binding specificity is confirmed by molecular docking [38, 39] (see **c**). Box and Violin plots are drawn by BoxPlotR [40] and PlotsOfData [41], respectively

Next, we verified whether the top 100 molecules are specific to Raf kinases (Group A) only. Figure 5b shows that, even though sequence and structure homology of the kinase domains are high, the binding of the top 100 molecules is specific to Raf kinases, but not to other kinases (Group B and D). Group B kinases are known to be inhibited by sorafenib. However, the top 100 molecules show higher binding affinity to Raf kinases than that of Group B kinases. This result was further confirmed by structure-based molecular docking analysis, which showed that the top 100 molecules exhibit more favorable interaction with B-RAF (Group A) than with Group B or Group D (Fig. 5c). Taken together, these results indicate that the proposed model can significantly reduce off-target drug interaction.

**Fig. 6:**
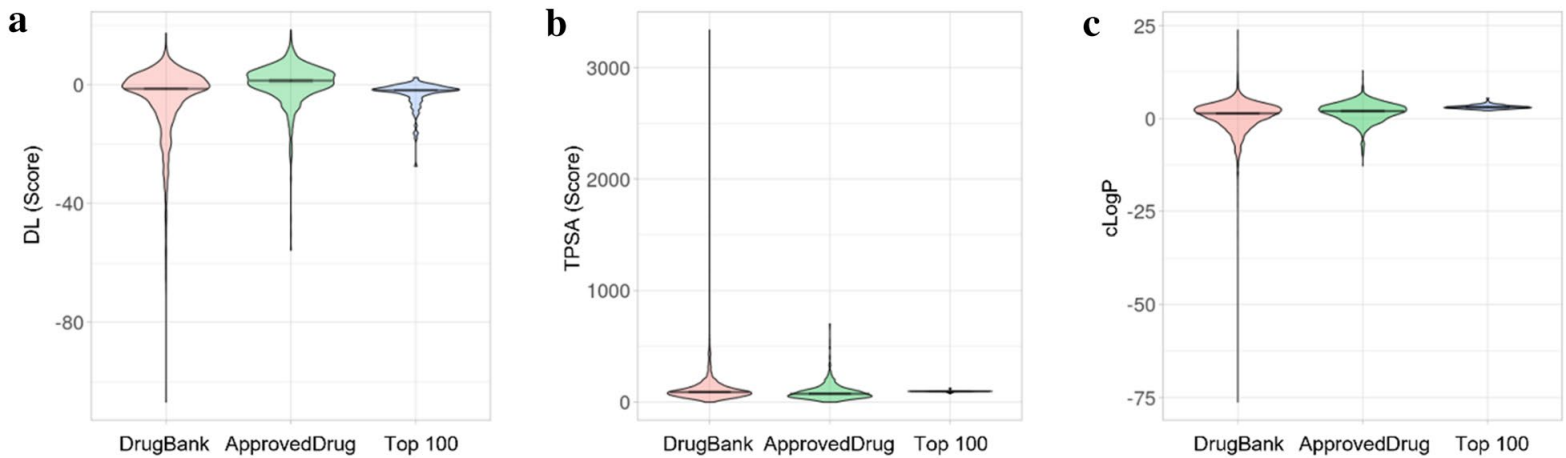
Comparison of druggability: Distribution of Druglikeness (**a**), Topological polar surface area (TPSA) (**b**), and Calculated logP (**c**) of DrugBank drugs, FDA-approved drugs, and the top 100 molecules. Violin plot drawn by PlotsOfData

We also found out whether the top 100 molecules are druggable. Figure 6a, b and c show that Druglikeness (DL), topological polar surface area (TPSA), and calculated logP (cLogP) of the top 100 molecules are comparable to those of pre-existing DrugBank and FDA-approved drugs.

**Fig. 7:**
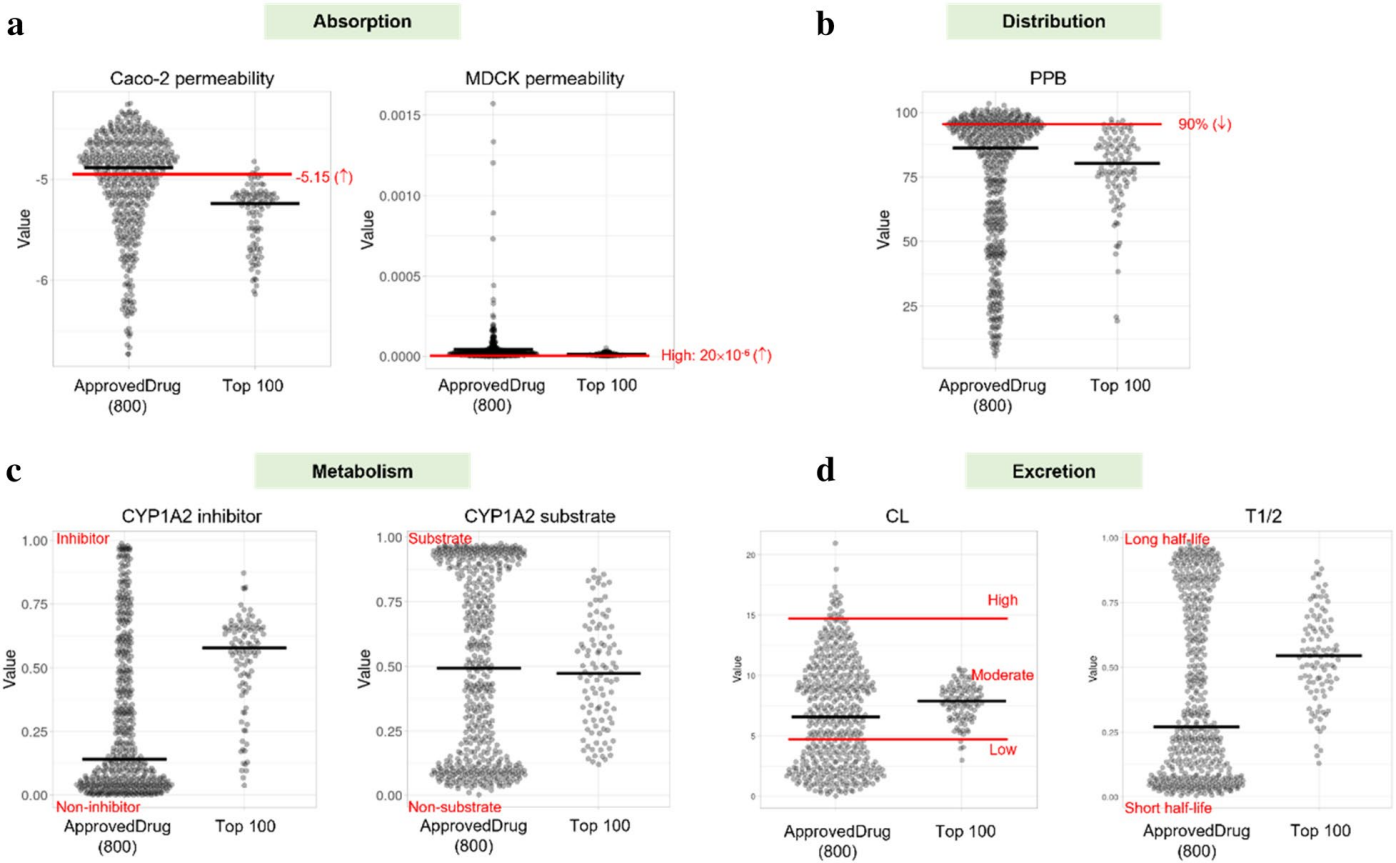
In silico ADME profiles: Profiles of Absorption (**a**), Distribution (**b**), Metabolism (**c**), and Excretion (**d**). Violin plot drawn by PlotsOfData

We evaluated whether the top 100 molecules have more desirable ADME (Absorption, Distribution, Metabolism, and Excretion) profiles when compared with FDA-approved drugs using ADMETlab2.0 [42]. Among 23 classified models, seven representative models were included in Fig. 7. The overall ADME profile of the top 100 molecules shows similarity to FDA-approved drugs. Specifically, the top 100 molecules have distribution and excretion profiles similar to those of FDA-approved drugs. Furthermore, their evaluation scores for distribution and excretion are excellent (Fig. 7b and d). On the other hand, the evaluation score for absorption and metabolism is moderate and slightly poor, respectively (Fig. 7a and 7c).

**Fig. 8:**
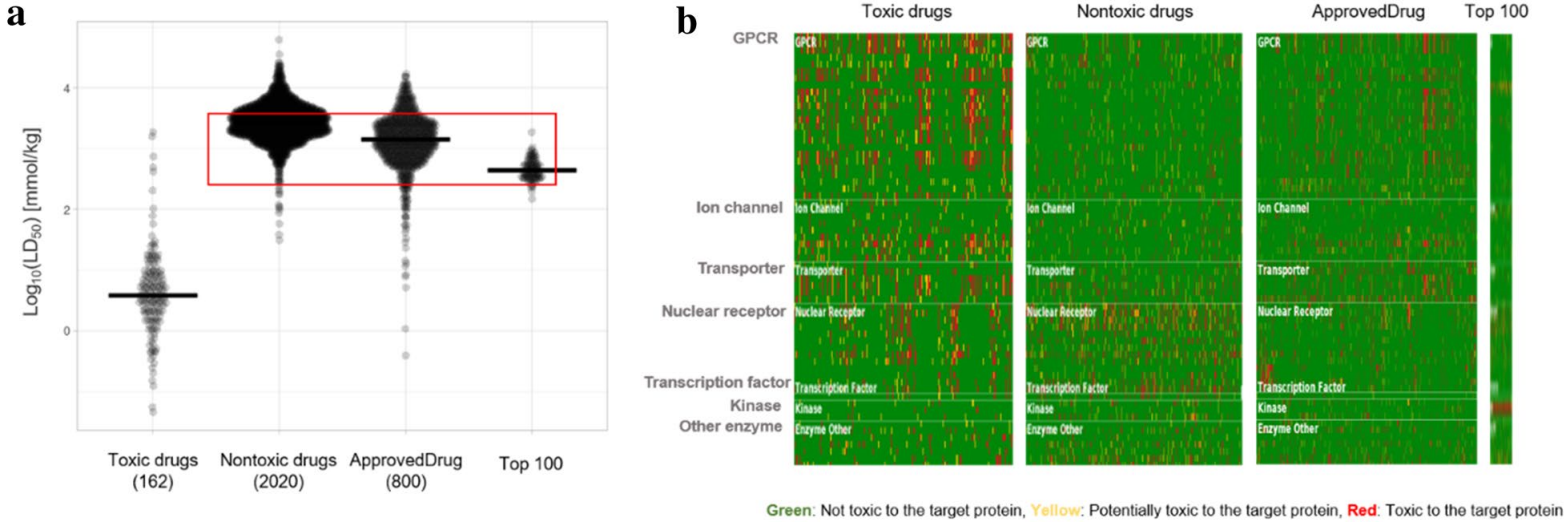
In silico toxicity profiles: Acute oral toxicity (median lethal death, LD_50_) prediction was calculated by Xu et al. [45] (see **a**). In silico pharmacological profiling and assessment of the potential for off-target interactions of drugs are shown in **b**. Heatmap and violin plot are drawn by PlotsOfData and ToxProfiler, respectively

We also performed in silico toxicity profiling analysis. Toxicity data of chemical molecules were obtained from Gadaleta et al. [43]. Figure 8a shows that the chemical acute toxicity of the top 100 molecules was a little higher than non-toxic drugs and FDA-approved drugs, but still very much lower than toxic drugs. Figure 8b shows in silico pharmacological profiling and assessment of the potential interaction for 64 toxicity off-targets, and we can observe that the profiles of the top 100 molecules are similar to non-toxic drugs rather than to toxic drugs. In silico pharmacological profiles were created by ToxProfiler [44]. Figures 6, 7, and 8 all show that the top 100 molecules are highly druggable.

**Fig. 9:**
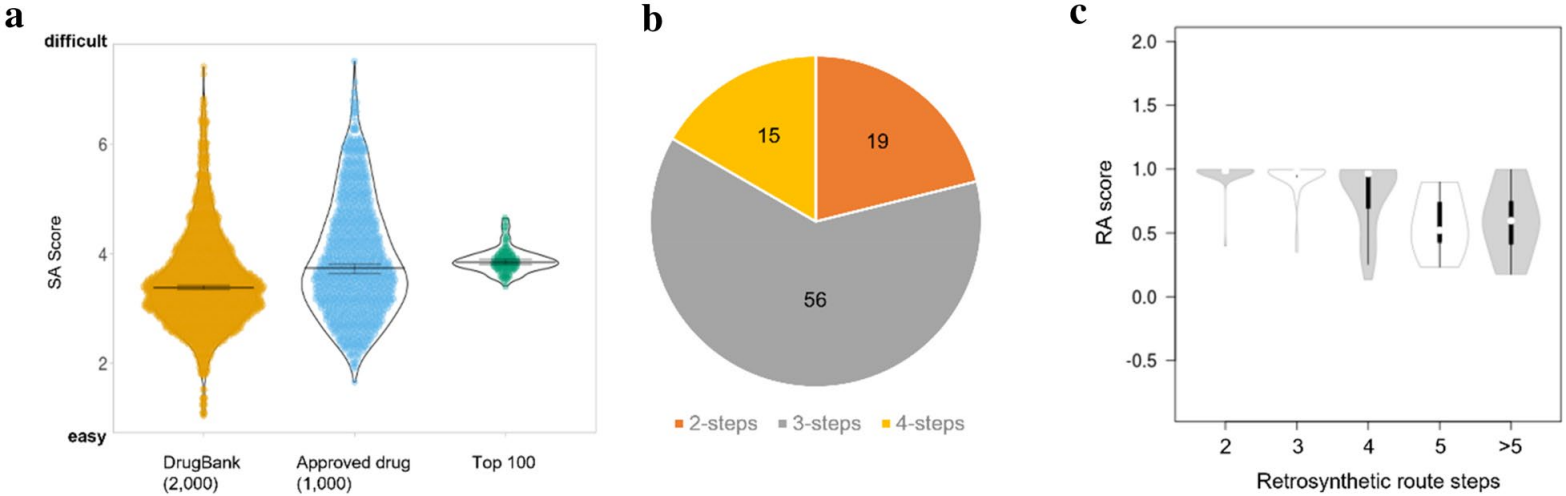
Comparison of synthesizability: Comparison of SA score (**a**). Feasibility of compound synthesis by solved retrosynthetic routes of top 100 molecules [47] (**b**). Correlation analysis between retrosynthetic steps and the RA score (**c**). Violin and box plots were drawn by PlotsOfData and BoxPlotR, respectively

Finally, we checked whether the top 100 molecules are synthesizable. Figure 9a shows that the Synthetic Accessibility (SA) scores of the top 100 molecules are slightly higher than those of DrugBank drugs and FDA-approved drugs; nevertheless, the SA scores of the top 100 molecules exist within the acceptable range based upon previous publications [46]. Drugs for comparison were selected according to their molecular weight (300 < MW < 600). Figure 9b shows that 19, 56, and 15 drugs among the top 100 molecules can be synthesized in 2, 3, and 4 steps, respectively, through solved retrosynthetic pathways [47]. Further analysis shows that the top 100 molecules with fewer steps have good RA scores (Fig. 9c). Taken together, these results suggest that 90 out of 100 molecules can be synthesized using chemical reactions.

## Conclusions

For this study, we proposed a reinforcement learning model that could maximize the predicted binding affinity between a generated molecule and target proteins, while existing methods mainly consider the chemical properties of the created molecules. The agent of the proposed reinforcement learning model is Stack-CVAE, and policy is a probability distribution of the decoder of stack-CVAE. The rewards are measured using the synthesizability of the generated molecules and the binding affinity between the generated molecules and the target proteins, and the model is trained to increase the rewards.

After the model was trained, we generated 1000 chemical formulas using chemical properties and the three Raf target proteins of sorafenib. The proportion of valid and unique chemical compounds of Stack-CVAE is higher than that of other generative models. The proportion of chemical compounds that have the desired chemical properties (such as molecular weight, logP, TPSA, and RAscore) and higher binding affinity of Stack-CVAE is also higher. Further quantitative and qualitative analysis of the top 100 scoring molecules shows that they are novel and have higher binding affinity for Raf proteins than for other proteins. This 100 of the top scoring molecules are also highly druggable and synthesizable.

## Supplementary Information

**Additional file 1: Figure S1.** Top 100 SMILES.

## Data Availability

https://github.com/HwanheeKim813/stack_CVAE.git, DrugBank, https://www.drugbank.ca/releases/latest, ChEMBL, https://www.ebi.ac.uk/chembl/

## Abbreviations

AE: AutoEncoder
CADD: Computer Aided Drug Discovery
CVAE: Conditional AutoEncoder
DL: Druglikeness
TPSA: Topological polar surface
SA: Synthetic accessibiliry
DNN: Deep Neural Network
DTBA: Drug-Target Binding Affinity
LBDD: Ligand-Based Drug Design
LSTM: Long Short-Term Memory
S2SAE: Sequence-To-Sequence Auto-Encoder
SBDD: Structure-Based Drug Design
SMILES: Simplified Molecular Input Line Entry System
Stack-RNN: Stack augmented RNN
stack-CVAE: Conditional variational autoencoder with Stack augmented RNN
RAscore: Retrosynthetic accessibility score
RNN: Recurrent Neural Network
GAN: Generative Adversarial Network
GRU: Gated Recurrent Units
VAE: Variational AutoEncoder
