# Supplementary material for: Predicting chemical structure using reinforcement learning with a stack-augmented conditional variational autoencoder
Source: J Cheminform. 2022 Dec 9;14:83. doi: 10.1186/s13321-022-00666-9 (PMC9733204; doi:10.1186/s13321-022-00666-9)
Supplement: Supplementary file 1 — Additional file 1: Figure S1. Top 100 SMILES. [file 13321_2022_666_MOESM1_ESM.docx]

**Supplementary fig. S1. Top 100 SMILES**

1) CCOCNc1ncccc1-c1cccnc1NCc1cccnc1NCCCCCCOF


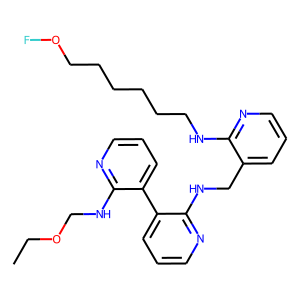


Molecular Weight: 468.26 LogP: 5.43 TPSA: 93.22
Target Binding affinity: 7.72 GroupD Binding affinity: 5.12 RAscore: 0.99

2) CCCCCCNc1ncccc1CNc1ncccc1CNc1ncccc1COCOC


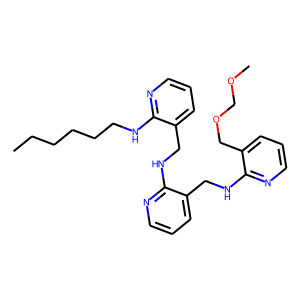


Molecular Weight: 464.29 LogP: 5.21 TPSA: 93.22
Target Binding affinity: 7.64 GroupD Binding affinity: 5.12 RAscore: 0.99

3) CCOCNc1nccnc1NCc1ccccc1NCc1cccnc1NC(C)CC(C)C


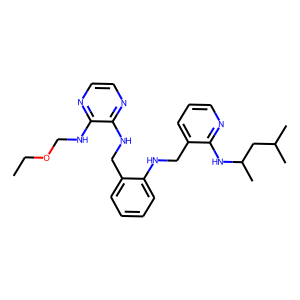


Molecular Weight: 463.31 LogP: 5.35 TPSA: 96.02
Target Binding affinity: 7.63 GroupD Binding affinity: 5.10 RAscore: 0.96

4) CCOCNc1ncccc1CNc1ncccc1CNc1ncccc1NCc1ccccc1


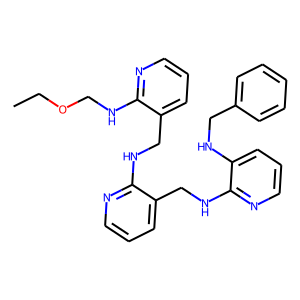


Molecular Weight: 469.26 LogP: 5.11 TPSA: 96.02
Target Binding affinity: 7.67 GroupD Binding affinity: 5.09 RAscore: 0.99

5) CCOCCNc1ncccc1CNc1ncccc1CNc1ncccc1Oc1ccccc1


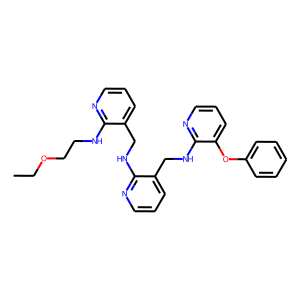


Molecular Weight: 470.24 LogP: 5.34 TPSA: 93.22
Target Binding affinity: 7.70 GroupD Binding affinity: 5.13 RAscore: 0.99

6) O=C(O)c1cccnc1NCc1cccnc1OCCNCCCCCCCCCCCF


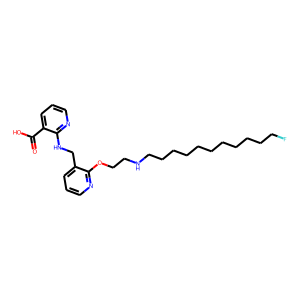


Molecular Weight: 460.28 LogP: 5.24 TPSA: 96.37
Target Binding affinity: 7.55 GroupD Binding affinity: 5.09 RAscore: 1.00

7) CCOCNc1ncccc1-c1nccnc1NCCCCOc1ccccc1-c1ccncc1


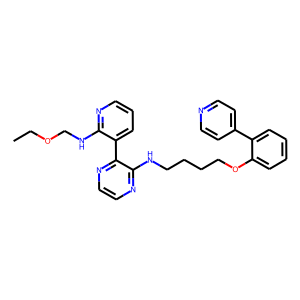


Molecular Weight: 470.24 LogP: 5.28 TPSA: 94.08
Target Binding affinity: 7.50 GroupD Binding affinity: 5.09 RAscore: 0.99

8) CCOCCNc1ncccc1CNc1ncccc1CNc1ncccc1-c1ccncc1C


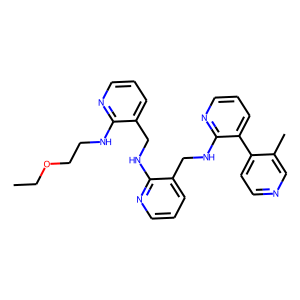


Molecular Weight: 469.26 LogP: 4.91 TPSA: 96.88
Target Binding affinity: 7.80 GroupD Binding affinity: 5.09 RAscore: 0.98

9) CCOCCCNc1ncncc1CNc1ncccc1-c1cccnc1NCc1ccccc1


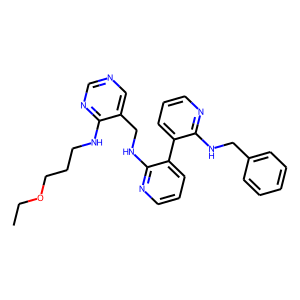


Molecular Weight: 469.26 LogP: 5.00 TPSA: 96.88
Target Binding affinity: 7.74 GroupD Binding affinity: 5.10 RAscore: 0.99

10) C#CNCc1cccnc1NCc1cccnc1NCc1ccccc1Oc1ccccc1C#N


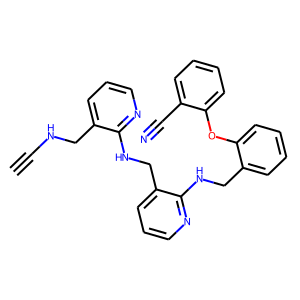


Molecular Weight: 460.20 LogP: 5.05 TPSA: 94.89
Target Binding affinity: 7.58 GroupD Binding affinity: 5.09 RAscore: 0.99

11) CCCCCNc1ncccc1CNc1ncccc1CNc1ncccc1C(C)OOC


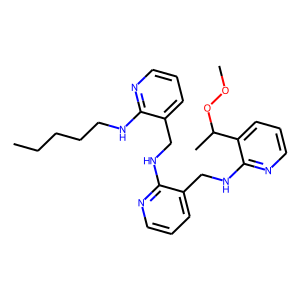


Molecular Weight: 450.27 LogP: 5.34 TPSA: 93.22
Target Binding affinity: 7.75 GroupD Binding affinity: 5.11 RAscore: 0.99

12) CCOCNc1ncccc1-c1cccnc1NCc1cccnc1NCc1cccnc1F


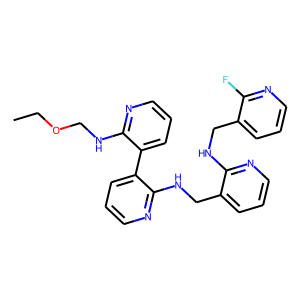


Molecular Weight: 459.22 LogP: 4.70 TPSA: 96.88
Target Binding affinity: 7.72 GroupD Binding affinity: 5.08 RAscore: 0.98

13) CCOCCNCc1cccnc1NCc1cccnc1NCc1ccccc1-c1ccoc1


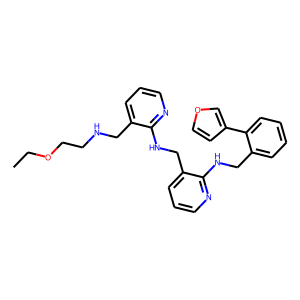


Molecular Weight: 457.25 LogP: 5.09 TPSA: 84.24
Target Binding affinity: 7.62 GroupD Binding affinity: 5.07 RAscore: 0.99

14) CCOCCNc1nccnc1NCc1cccnc1NCc1ccccc1COC(C)(C)C


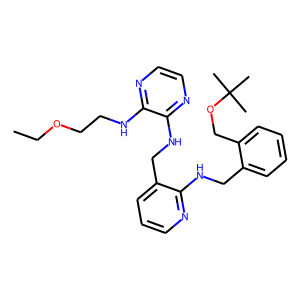


Molecular Weight: 464.29 LogP: 4.86 TPSA: 93.22
Target Binding affinity: 7.57 GroupD Binding affinity: 5.09 RAscore: 0.94

15) CCOCCCNc1ncccc1CNc1ncccc1-c1cccnc1NCc1ccco1


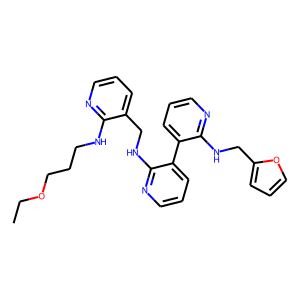


Molecular Weight: 458.24 LogP: 5.19 TPSA: 97.13
Target Binding affinity: 7.44 GroupD Binding affinity: 5.07 RAscore: 0.98

16) CCOCNc1ncccc1CNc1nccnc1NCc1ccccc1OCCCCCF


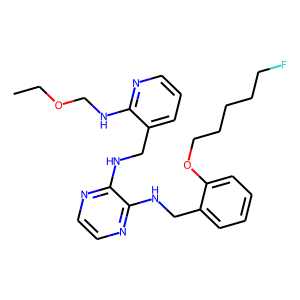


Molecular Weight: 468.26 LogP: 5.02 TPSA: 93.22
Target Binding affinity: 7.46 GroupD Binding affinity: 5.09 RAscore: 0.97

17) CCCCOCCNc1ncccc1CNc1nccnc1NCc1ccccc1COCF


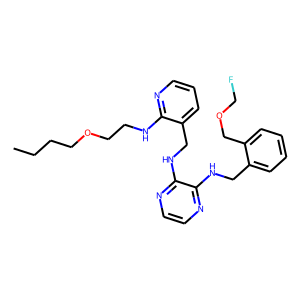


Molecular Weight: 468.26 LogP: 4.77 TPSA: 93.22
Target Binding affinity: 7.59 GroupD Binding affinity: 5.10 RAscore: 0.99

18) CCCCCCCC(=S)NNc1nccc(-c2cccnc2NCCc2ccncc2)n1


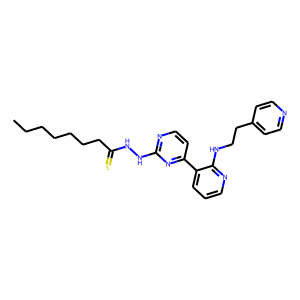


Molecular Weight: 449.24 LogP: 5.19 TPSA: 87.65
Target Binding affinity: 7.68 GroupD Binding affinity: 5.08 RAscore: 0.97

19) CCCOCCNc1ncccc1CNc1ncccc1CNc1ncccc1-c1ccncc1


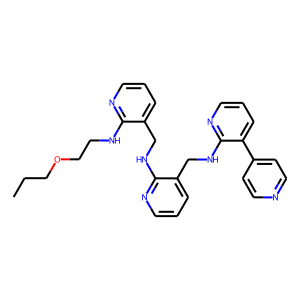


Molecular Weight: 469.26 LogP: 5.00 TPSA: 96.88
Target Binding affinity: 7.59 GroupD Binding affinity: 5.09 RAscore: 0.99

20) CCOCNc1ncccc1CNc1ncccc1-c1cccnc1NCc1ccncc1F


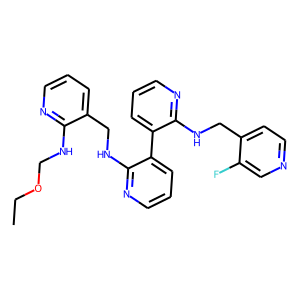


Molecular Weight: 459.22 LogP: 4.70 TPSA: 96.88
Target Binding affinity: 7.68 GroupD Binding affinity: 5.08 RAscore: 0.99

21) CCOCCCNc1ncccc1CNc1ncccc1CNc1ncccc1-c1cccnc1


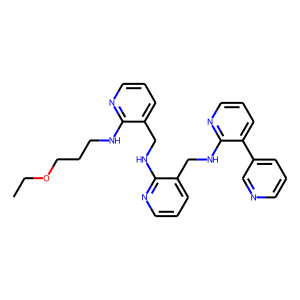


Molecular Weight: 469.26 LogP: 5.00 TPSA: 96.88
Target Binding affinity: 7.63 GroupD Binding affinity: 5.10 RAscore: 0.99

22) CCNCc1cccnc1NCc1cccnc1NCc1ccncc1Oc1ccccc1OC


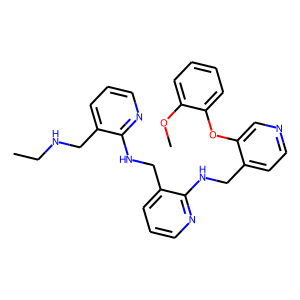


Molecular Weight: 470.24 LogP: 5.01 TPSA: 93.22
Target Binding affinity: 7.57 GroupD Binding affinity: 5.11 RAscore: 0.98

23) CCNc1ncccc1-c1ncncc1CNc1ncccc1CNc1ncccc1C(F)F


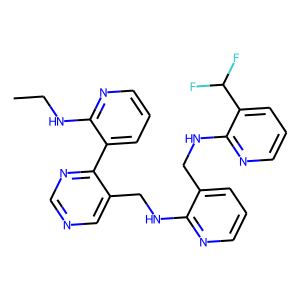


Molecular Weight: 462.21 LogP: 4.92 TPSA: 100.54
Target Binding affinity: 7.49 GroupD Binding affinity: 5.06 RAscore: 0.96

24) CCOCNc1ncccc1CNc1ncccc1-c1ccncc1CNc1ncccc1C


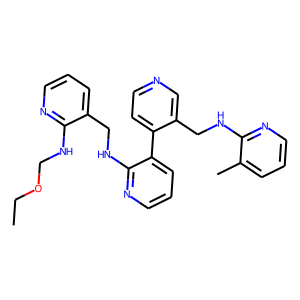


Molecular Weight: 455.24 LogP: 4.87 TPSA: 96.88
Target Binding affinity: 7.63 GroupD Binding affinity: 5.08 RAscore: 0.99

25) CCOCCNc1ncccc1CNc1ncccc1-c1cccnc1NCc1ccncc1


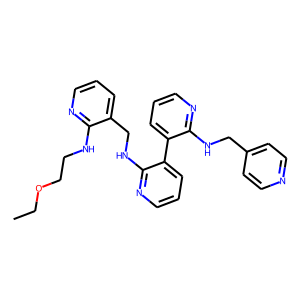


Molecular Weight: 455.24 LogP: 4.61 TPSA: 96.88
Target Binding affinity: 7.85 GroupD Binding affinity: 5.10 RAscore: 1.00

26) CCOCNc1ncccc1CNc1nccnc1NCc1ccccc1-c1ccncc1C


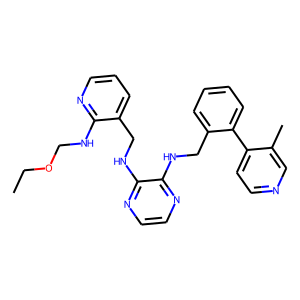


Molecular Weight: 455.24 LogP: 4.87 TPSA: 96.88
Target Binding affinity: 7.46 GroupD Binding affinity: 5.05 RAscore: 0.98

27) CCOCNc1ncccc1CNc1ncccc1CNc1ncccc1NCCC=C(C)C


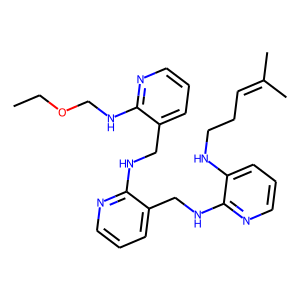


Molecular Weight: 461.29 LogP: 5.27 TPSA: 96.02
Target Binding affinity: 7.65 GroupD Binding affinity: 5.12 RAscore: 0.96

28) CCOCCNc1ncccc1CNc1ncccc1-c1cccnc1NCc1cccnc1


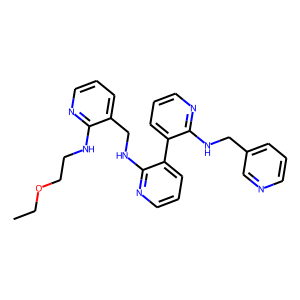


Molecular Weight: 455.24 LogP: 4.61 TPSA: 96.88
Target Binding affinity: 7.83 GroupD Binding affinity: 5.09 RAscore: 1.00

29) CCOCNc1nccnc1NCc1cccnc1NCc1ccccc1COc1ccccc1


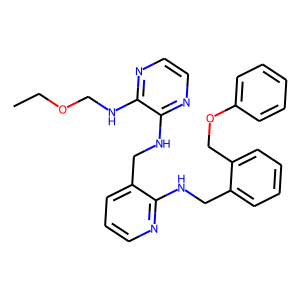


Molecular Weight: 470.24 LogP: 5.08 TPSA: 93.22
Target Binding affinity: 7.46 GroupD Binding affinity: 5.11 RAscore: 0.99

30) CCOCNc1ncccc1CNc1ncccc1CNc1ncccc1-c1cccnc1C


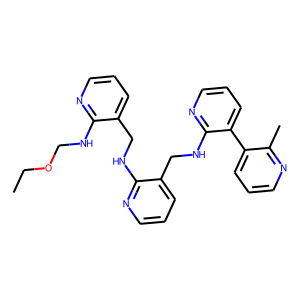


Molecular Weight: 455.24 LogP: 4.87 TPSA: 96.88
Target Binding affinity: 7.56 GroupD Binding affinity: 5.07 RAscore: 0.99

31) COCCNc1ncccc1CNc1ncccc1NCc1ccncc1NCc1ccccc1


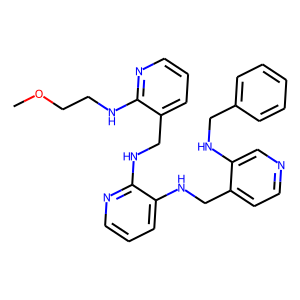


Molecular Weight: 469.26 LogP: 4.77 TPSA: 96.02
Target Binding affinity: 7.77 GroupD Binding affinity: 5.11 RAscore: 0.98

32) CCOCCCNc1ncccc1Cc1cccnc1NCc1ccccc1CCCCN=O


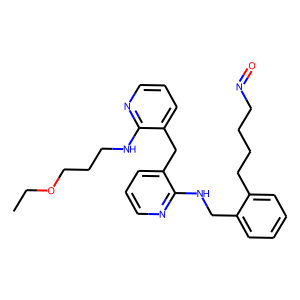


Molecular Weight: 461.28 LogP: 5.61 TPSA: 88.50
Target Binding affinity: 7.50 GroupD Binding affinity: 5.10 RAscore: 0.89

33) CCOCNc1ncccc1-c1cccnc1NCc1cccnc1NC=Cc1cccnc1


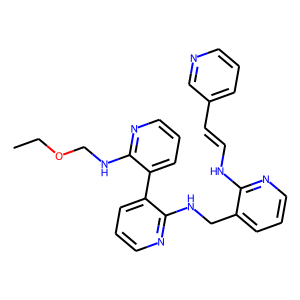


Molecular Weight: 453.23 LogP: 5.03 TPSA: 96.88
Target Binding affinity: 7.63 GroupD Binding affinity: 5.09 RAscore: 0.99

34) COCc1cccnc1NCc1cccnc1NCc1cccnc1NCc1ccccc1OC


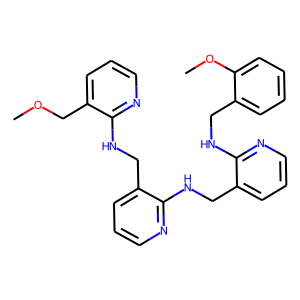


Molecular Weight: 470.24 LogP: 4.86 TPSA: 93.22
Target Binding affinity: 7.40 GroupD Binding affinity: 5.09 RAscore: 0.99

35) CCCCCCNc1ncccc1CNc1ncccc1CNc1ncccc1C(=O)OCF


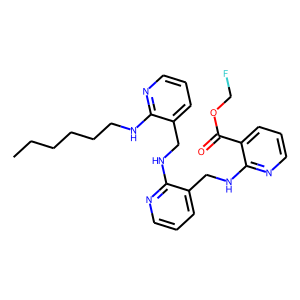


Molecular Weight: 466.25 LogP: 5.17 TPSA: 101.06
Target Binding affinity: 7.65 GroupD Binding affinity: 5.11 RAscore: 0.99

36) CCOCNc1ncccc1CNc1ncccc1-c1cccnc1NCc1ccncc1C


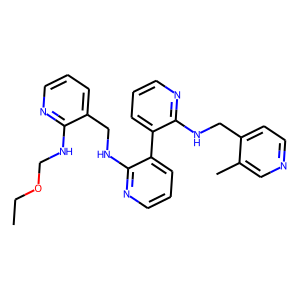


Molecular Weight: 455.24 LogP: 4.87 TPSA: 96.88
Target Binding affinity: 7.70 GroupD Binding affinity: 5.10 RAscore: 0.99

37) CCOCCNc1ncccc1CNc1ncccc1CNc1ncccc1-c1cccnc1


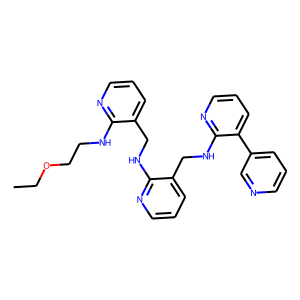


Molecular Weight: 455.24 LogP: 4.61 TPSA: 96.88
Target Binding affinity: 7.83 GroupD Binding affinity: 5.10 RAscore: 0.99

38) CCCCCNCc1cccnc1NCc1cccnc1NCc1cccnc1NC(C)OC


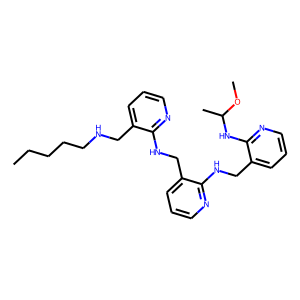


Molecular Weight: 463.31 LogP: 4.78 TPSA: 96.02
Target Binding affinity: 7.69 GroupD Binding affinity: 5.12 RAscore: 0.99

39) CCOCNc1ncccc1CNc1ncccc1-c1ncccc1NCc1ccccc1OC


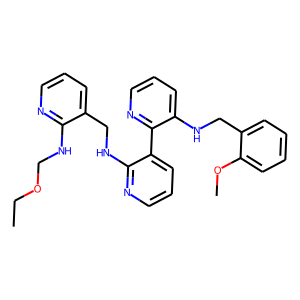


Molecular Weight: 470.24 LogP: 5.18 TPSA: 93.22
Target Binding affinity: 7.02 GroupD Binding affinity: 5.06 RAscore: 0.99

40) CCOCNc1ncccc1CNc1ncccc1CNc1ncccc1OCc1ccccc1


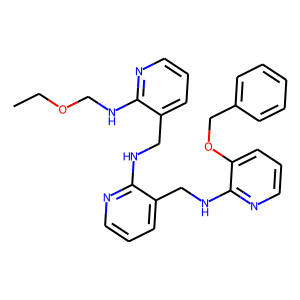


Molecular Weight: 470.24 LogP: 5.08 TPSA: 93.22
Target Binding affinity: 7.57 GroupD Binding affinity: 5.13 RAscore: 0.99

41) CCOCCNc1ncccc1CNc1ncccc1CNc1ccncc1NCCC(C)C


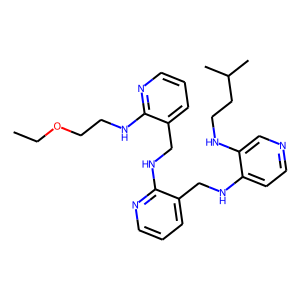


Molecular Weight: 463.31 LogP: 5.00 TPSA: 96.02
Target Binding affinity: 7.63 GroupD Binding affinity: 5.13 RAscore: 0.99

42) CCOCNc1ncccc1CNc1ncccc1CNc1ncccc1-c1ccncc1C


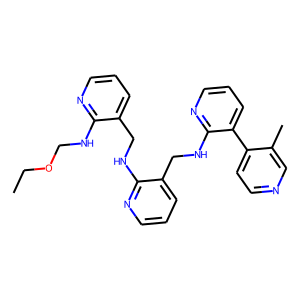


Molecular Weight: 455.24 LogP: 4.87 TPSA: 96.88
Target Binding affinity: 7.62 GroupD Binding affinity: 5.08 RAscore: 0.98

43) CCCCNc1ncccc1CNc1ncccc1CNc1ncccc1NCc1cccnc1


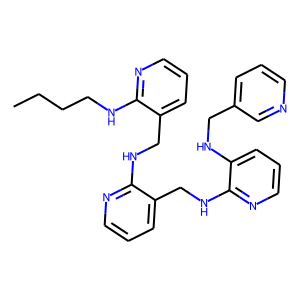


Molecular Weight: 468.27 LogP: 5.31 TPSA: 99.68
Target Binding affinity: 7.47 GroupD Binding affinity: 5.10 RAscore: 0.99

44) CCOCCNc1ncccc1CNc1ncccc1CNc1ncccc1CCc1ccco1


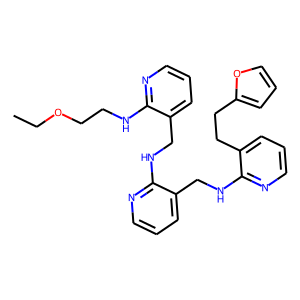


Molecular Weight: 472.26 LogP: 4.92 TPSA: 97.13
Target Binding affinity: 7.77 GroupD Binding affinity: 5.12 RAscore: 0.99

45) CCOCNc1ncccc1CNc1ncccc1-c1cccnc1NC(=S)OCCCF


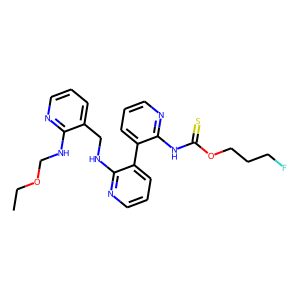


Molecular Weight: 470.19 LogP: 4.63 TPSA: 93.22
Target Binding affinity: 7.57 GroupD Binding affinity: 5.10 RAscore: 0.99

46) C#CNCc1ccccc1CNc1ncccc1CNc1ncccc1-c1ccccc1CN=N


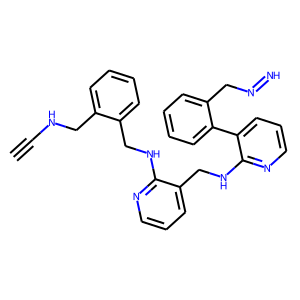


Molecular Weight: 461.23 LogP: 5.58 TPSA: 98.08
Target Binding affinity: 7.50 GroupD Binding affinity: 5.08 RAscore: 0.85

47) CCOCCCNc1ncccc1CNc1ncccc1CNc1ncccc1-c1ccncc1


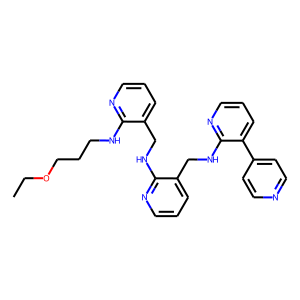


Molecular Weight: 469.26 LogP: 5.00 TPSA: 96.88
Target Binding affinity: 7.50 GroupD Binding affinity: 5.10 RAscore: 0.99

48) CCNCc1cccnc1NCc1cccnc1Nc1ncccc1NCc1ccccc1OC


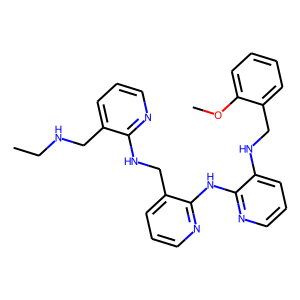


Molecular Weight: 469.26 LogP: 4.96 TPSA: 96.02
Target Binding affinity: 7.32 GroupD Binding affinity: 5.08 RAscore: 0.99

49) CCOCNc1ncccc1CNc1ncccc1CNc1ncccc1-c1cccnc1F


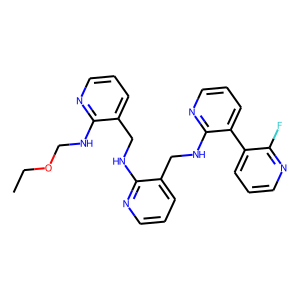


Molecular Weight: 459.22 LogP: 4.70 TPSA: 96.88
Target Binding affinity: 7.60 GroupD Binding affinity: 5.09 RAscore: 0.98

50) CCCCCNN=Cc1ccccc1CNc1ncccc1CNc1ncccc1C(=O)CF


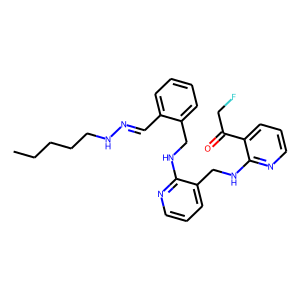


Molecular Weight: 462.25 LogP: 4.97 TPSA: 91.30
Target Binding affinity: 7.27 GroupD Binding affinity: 5.09 RAscore: 0.99

51) CCNc1ncncc1-c1cccnc1NCc1cccnc1NCc1ccccc1COF


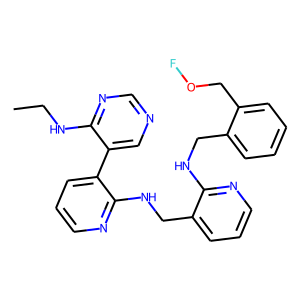


Molecular Weight: 459.22 LogP: 4.99 TPSA: 96.88
Target Binding affinity: 7.60 GroupD Binding affinity: 5.10 RAscore: 0.97

52) CCOc1cc(O)ccc1CNc1ncccc1-c1cccnc1NCc1cccnc1CF


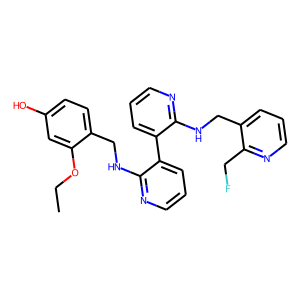


Molecular Weight: 459.21 LogP: 5.34 TPSA: 92.19
Target Binding affinity: 6.83 GroupD Binding affinity: 5.05 RAscore: 0.99

53) CCOCCNc1ncccc1CNc1nnccc1NCc1ccccc1-c1cccnc1


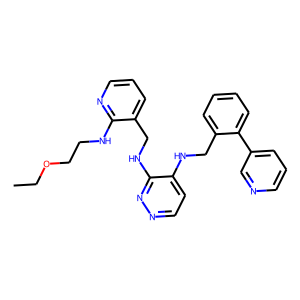


Molecular Weight: 455.24 LogP: 4.61 TPSA: 96.88
Target Binding affinity: 7.52 GroupD Binding affinity: 5.06 RAscore: 0.99

54) CCCNc1ncccc1CNc1ncccc1CNc1nccnc1CCCOC(C)CC


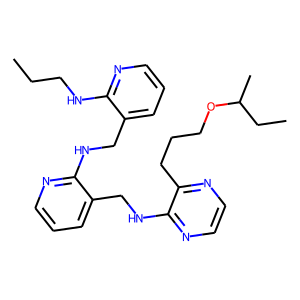


Molecular Weight: 463.31 LogP: 5.06 TPSA: 96.88
Target Binding affinity: 7.55 GroupD Binding affinity: 5.12 RAscore: 0.99

55) CCCCNCc1cccnc1Nc1ncccc1CNc1ncccc1-c1cnccc1OC


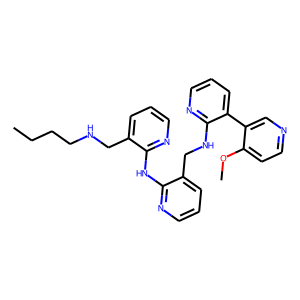


Molecular Weight: 469.26 LogP: 5.19 TPSA: 96.88
Target Binding affinity: 7.34 GroupD Binding affinity: 5.09 RAscore: 0.99

56) CCCCCn1ccnc1NCc1cccnc1NCc1cccnc1NCc1ccncc1


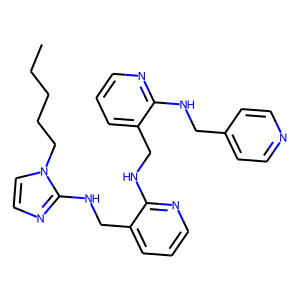


Molecular Weight: 456.27 LogP: 5.09 TPSA: 92.58
Target Binding affinity: 7.47 GroupD Binding affinity: 5.12 RAscore: 1.00

57) CCCCNCc1ccccc1CNc1ncccc1CNc1ncccc1CNC(C)(C)O


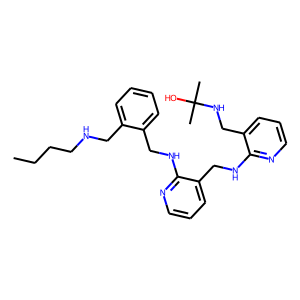


Molecular Weight: 462.31 LogP: 4.41 TPSA: 94.13
Target Binding affinity: 7.48 GroupD Binding affinity: 5.08 RAscore: 0.99

58) CCOCNc1ncccc1CNc1ncccc1CNc1ncccc1Nc1ccccc1


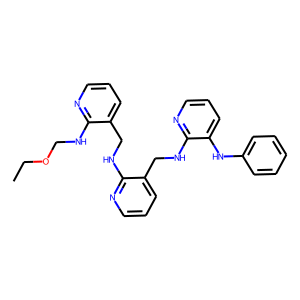


Molecular Weight: 455.24 LogP: 5.25 TPSA: 96.02
Target Binding affinity: 7.70 GroupD Binding affinity: 5.13 RAscore: 0.98

59) CCCCCNCc1ccccc1CNc1ncccc1CNc1ncccc1CNOOC


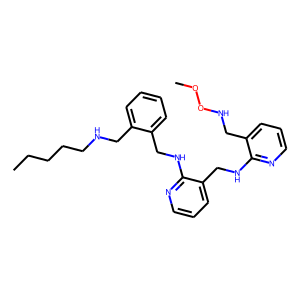


Molecular Weight: 464.29 LogP: 4.56 TPSA: 92.36
Target Binding affinity: 7.33 GroupD Binding affinity: 5.09 RAscore: 1.00

60) CCOCNc1ncccc1CNc1ncccc1CNc1ncccc1COc1ccccc1


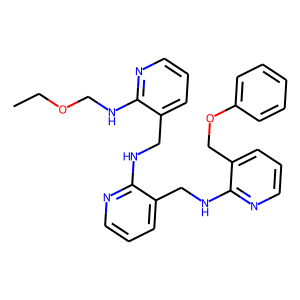


Molecular Weight: 470.24 LogP: 5.08 TPSA: 93.22
Target Binding affinity: 7.62 GroupD Binding affinity: 5.14 RAscore: 0.99

61) CCOCNc1ncccc1CNc1ncccc1-c1cccnc1NCc1cccnc1C


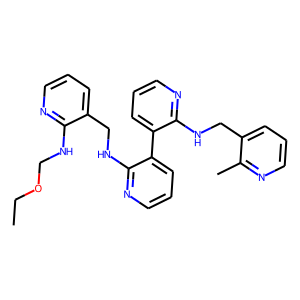


Molecular Weight: 455.24 LogP: 4.87 TPSA: 96.88
Target Binding affinity: 7.57 GroupD Binding affinity: 5.08 RAscore: 0.97

62) CCOCNc1ncccc1CNc1ncccc1CNc1ncccc1Oc1ccccc1


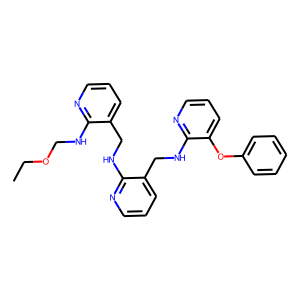


Molecular Weight: 456.23 LogP: 5.29 TPSA: 93.22
Target Binding affinity: 7.50 GroupD Binding affinity: 5.13 RAscore: 0.99

63) CCOCCNc1ncccc1CNc1ncccc1-c1cccnc1NCCCCOC


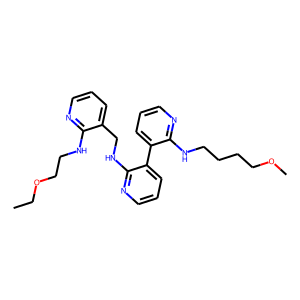


Molecular Weight: 450.27 LogP: 4.44 TPSA: 93.22
Target Binding affinity: 7.71 GroupD Binding affinity: 5.09 RAscore: 0.99

64) CCCOCNc1ncncc1CNc1ncccc1CNc1ncccc1-c1ccccc1C


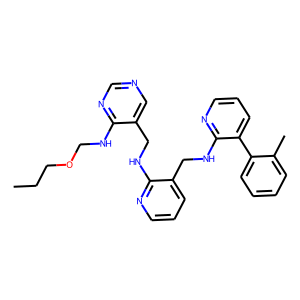


Molecular Weight: 469.26 LogP: 5.26 TPSA: 96.88
Target Binding affinity: 7.54 GroupD Binding affinity: 5.12 RAscore: 0.98

65) CCOCCCNc1ncccc1CNc1ncccc1CNc1ncccc1COCCC


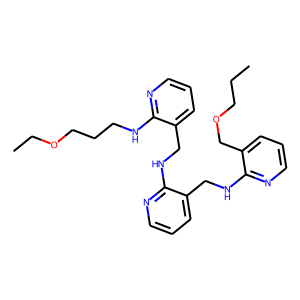


Molecular Weight: 464.29 LogP: 4.86 TPSA: 93.22
Target Binding affinity: 7.39 GroupD Binding affinity: 5.11 RAscore: 0.99

66) CCCCCCCNc1ncccc1CNc1nnccc1NCCCCNCCCCOC


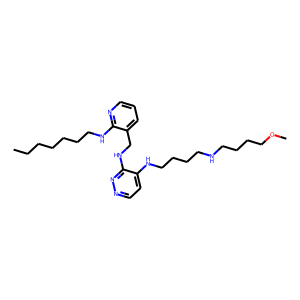


Molecular Weight: 471.37 LogP: 5.07 TPSA: 96.02
Target Binding affinity: 7.40 GroupD Binding affinity: 5.10 RAscore: 0.99

67) CCOCNc1ncccc1CNc1ncccc1CNc1ncccc1Cc1cccnc1


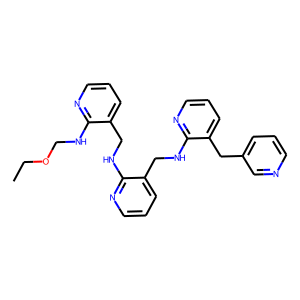


Molecular Weight: 455.24 LogP: 4.49 TPSA: 96.88
Target Binding affinity: 7.86 GroupD Binding affinity: 5.11 RAscore: 0.99

68) CCCCOc1ncccc1CNc1ncccc1CNc1nnccc1OCC(C)C(C)C


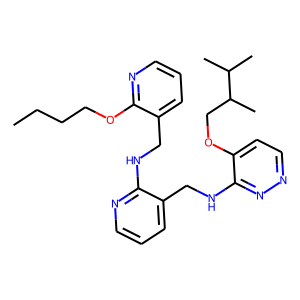


Molecular Weight: 464.29 LogP: 5.34 TPSA: 94.08
Target Binding affinity: 7.22 GroupD Binding affinity: 5.12 RAscore: 0.98

69) CCOCCNc1nccnc1NCc1cccnc1NCc1ccccc1-c1ccccc1


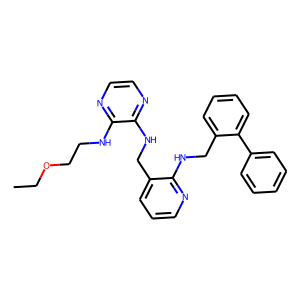


Molecular Weight: 454.25 LogP: 5.21 TPSA: 83.99
Target Binding affinity: 7.54 GroupD Binding affinity: 5.09 RAscore: 0.99

70) CCNCc1ncccc1NCc1cccnc1NCc1cccnc1NCCCCCCF


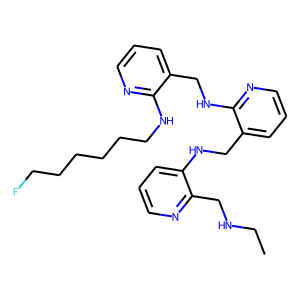


Molecular Weight: 465.30 LogP: 5.15 TPSA: 86.79
Target Binding affinity: 7.18 GroupD Binding affinity: 5.08 RAscore: 0.99

71) CCOCCNc1ncccc1-c1cccnc1NCc1cccnc1NCc1ccncc1


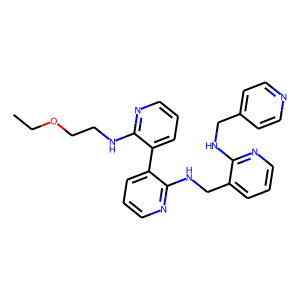


Molecular Weight: 455.24 LogP: 4.61 TPSA: 96.88
Target Binding affinity: 7.68 GroupD Binding affinity: 5.09 RAscore: 1.00

72) CC#CCCCCCCNc1ncccc1CNc1ncccc1CNCc1cnccc1O


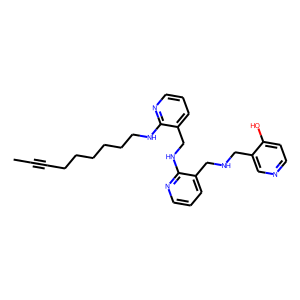


Molecular Weight: 458.28 LogP: 4.86 TPSA: 94.99
Target Binding affinity: 7.69 GroupD Binding affinity: 5.13 RAscore: 0.99

73) CCOCNc1ncccc1CNc1ncccc1-c1ncccc1CNc1ccncc1C


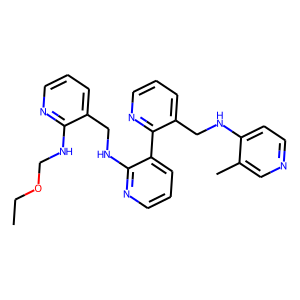


Molecular Weight: 455.24 LogP: 4.87 TPSA: 96.88
Target Binding affinity: 7.54 GroupD Binding affinity: 5.09 RAscore: 0.99

74) CCOCNc1ncncc1CNc1ncccc1-c1cccnc1NCc1ccccc1C


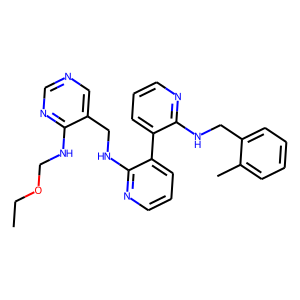


Molecular Weight: 455.24 LogP: 4.87 TPSA: 96.88
Target Binding affinity: 7.68 GroupD Binding affinity: 5.11 RAscore: 0.99

75) CCOCNCc1cccnc1NCc1cccnc1Nc1ncccc1COc1ccccc1


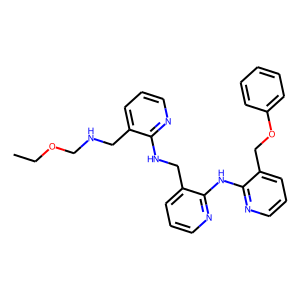


Molecular Weight: 470.24 LogP: 4.89 TPSA: 93.22
Target Binding affinity: 7.53 GroupD Binding affinity: 5.12 RAscore: 1.00

76) CCOCc1nccnc1Nc1ncccc1CNc1ncccc1-c1ncccc1C(F)F


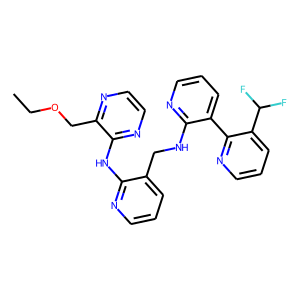


Molecular Weight: 463.19 LogP: 5.16 TPSA: 97.74
Target Binding affinity: 7.18 GroupD Binding affinity: 5.08 RAscore: 0.97

77) CCOCCNc1ncncc1-c1ccccc1NCc1cccnc1NCc1cccnc1


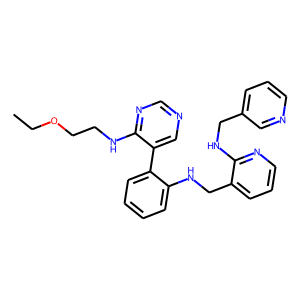


Molecular Weight: 455.24 LogP: 4.61 TPSA: 96.88
Target Binding affinity: 7.71 GroupD Binding affinity: 5.10 RAscore: 0.99

78) COCCCNc1ncccc1CNc1ncccc1CNc1ncccc1-c1ccccc1


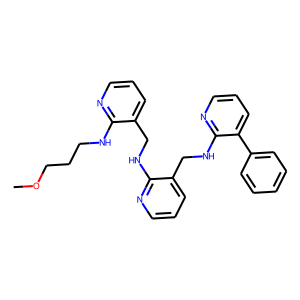


Molecular Weight: 454.25 LogP: 5.21 TPSA: 83.99
Target Binding affinity: 7.62 GroupD Binding affinity: 5.10 RAscore: 0.99

79) COCCCNc1ncccc1CNc1ncccc1CNc1ncccc1NCCC(C)C


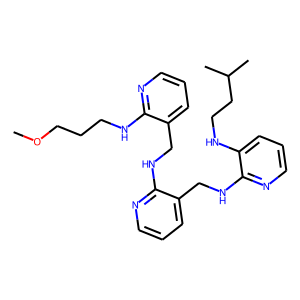


Molecular Weight: 463.31 LogP: 5.00 TPSA: 96.02
Target Binding affinity: 7.51 GroupD Binding affinity: 5.13 RAscore: 0.99

80) CCCN(Cc1cccnc1NCc1ccccc1NCc1cccnc1NC(C)(C)O)OC


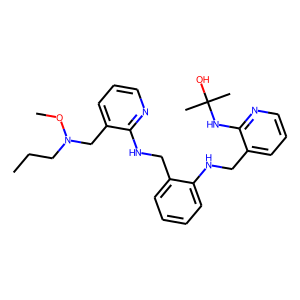


Molecular Weight: 464.29 LogP: 4.61 TPSA: 94.57
Target Binding affinity: 7.39 GroupD Binding affinity: 5.09 RAscore: 0.99

81) CCOCNc1ncccc1CNc1ncccc1-c1cccnc1NCCOC(C)CF


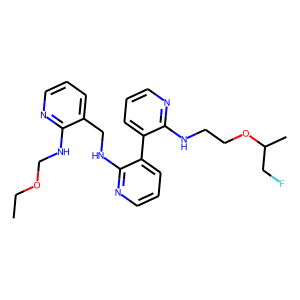


Molecular Weight: 454.25 LogP: 4.34 TPSA: 93.22
Target Binding affinity: 7.67 GroupD Binding affinity: 5.09 RAscore: 0.98

82) COCCNc1ncccc1CNc1ncccc1CNc1ccncc1Oc1ccccc1


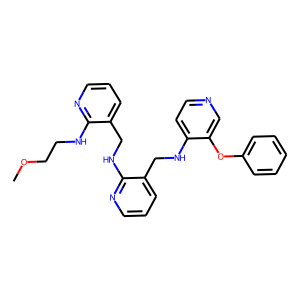


Molecular Weight: 456.23 LogP: 4.95 TPSA: 93.22
Target Binding affinity: 7.59 GroupD Binding affinity: 5.13 RAscore: 0.99

83) CCCCNc1ncccc1CNc1nccnc1NCc1nccn1Cc1ccccc1C


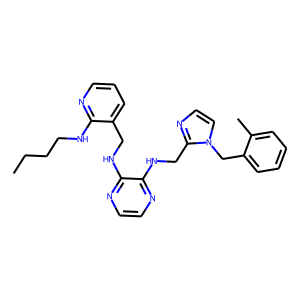


Molecular Weight: 456.27 LogP: 4.86 TPSA: 92.58
Target Binding affinity: 7.24 GroupD Binding affinity: 5.08 RAscore: 0.99

84) CCON=Cc1ccccc1CNc1cccnc1NCc1cccnc1NCc1cccnc1


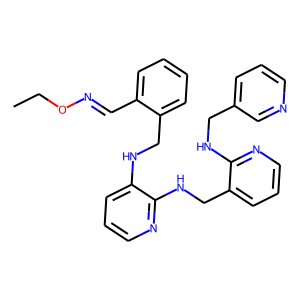


Molecular Weight: 467.24 LogP: 5.08 TPSA: 96.35
Target Binding affinity: 7.14 GroupD Binding affinity: 5.08 RAscore: 0.99

85) CCOCCNc1nccnc1NC(=O)c1ccccc1NCc1ccccc1-c1ccccc1


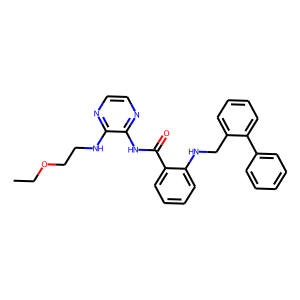


Molecular Weight: 467.23 LogP: 5.46 TPSA: 88.17
Target Binding affinity: 6.81 GroupD Binding affinity: 5.05 RAscore: 0.98

86) CCNc1cccnc1NCc1cccnc1NCc1cccnc1NCc1ccccc1OC


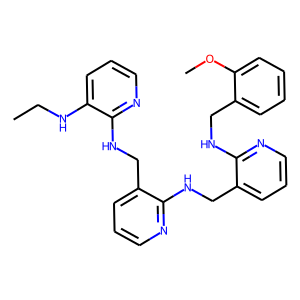


Molecular Weight: 469.26 LogP: 5.15 TPSA: 96.02
Target Binding affinity: 7.28 GroupD Binding affinity: 5.10 RAscore: 0.99

87) CCOCc1cccnc1NCc1cccnc1NCc1ccccc1-c1ccncc1CN


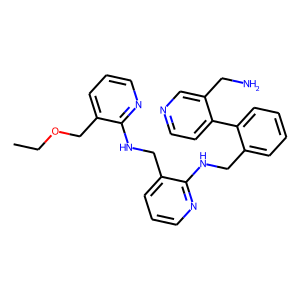


Molecular Weight: 454.25 LogP: 4.76 TPSA: 97.98
Target Binding affinity: 7.39 GroupD Binding affinity: 5.06 RAscore: 0.99

88) COCc1ccccc1CNc1ncccc1CNc1ncccc1NCc1ccccc1O


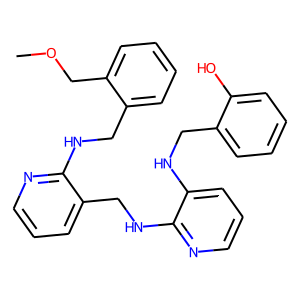


Molecular Weight: 455.23 LogP: 5.16 TPSA: 91.33
Target Binding affinity: 7.23 GroupD Binding affinity: 5.08 RAscore: 0.95

89) CCNCc1cccnc1CNc1ncccc1CNc1ncccc1-c1cccnc1C(C)C


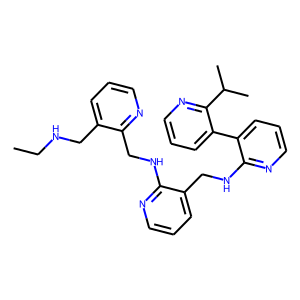


Molecular Weight: 467.28 LogP: 5.39 TPSA: 87.65
Target Binding affinity: 6.82 GroupD Binding affinity: 5.05 RAscore: 0.99

90) CCNc1ncccc1CNc1ncccc1-c1ccncc1CNc1ncccc1COC


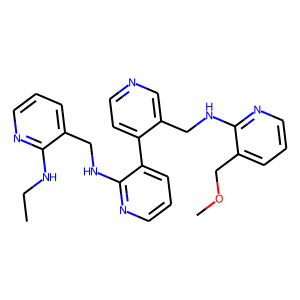


Molecular Weight: 455.24 LogP: 4.74 TPSA: 96.88
Target Binding affinity: 7.46 GroupD Binding affinity: 5.07 RAscore: 0.98

91) CCCCOC(=O)Nc1ncccc1CNc1nnccc1CCCc1ccccc1CF


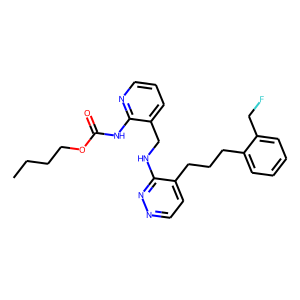


Molecular Weight: 451.24 LogP: 5.48 TPSA: 89.03
Target Binding affinity: 7.14 GroupD Binding affinity: 5.07 RAscore: 0.98

92) CCCCCC(N)C(C)OC(=O)NCc1ccccc1CNc1ncccc1CN1CCCC1


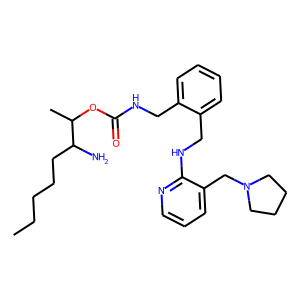


Molecular Weight: 467.33 LogP: 4.81 TPSA: 92.51
Target Binding affinity: 7.02 GroupD Binding affinity: 5.07 RAscore: 0.99

93) CCOCCNc1ncccc1CNc1ncccc1CNc1ncccc1-c1ccncc1


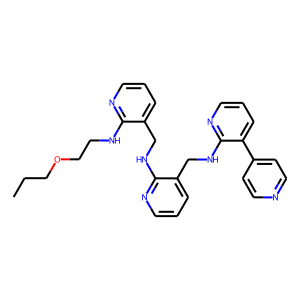


Molecular Weight: 455.24 LogP: 4.61 TPSA: 96.88
Target Binding affinity: 7.64 GroupD Binding affinity: 5.10 RAscore: 0.99

94) CCOc1ncccc1CNc1ncccc1-c1ncncc1CNc1ncccc1C(F)F


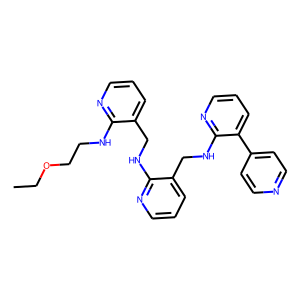


Molecular Weight: 463.19 LogP: 4.89 TPSA: 97.74
Target Binding affinity: 7.03 GroupD Binding affinity: 5.05 RAscore: 0.97

95) CCOCNc1ncccc1CNc1ncncc1CNc1ncccc1-c1ccccc1F


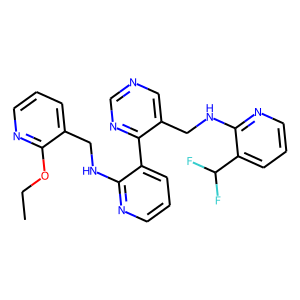


Molecular Weight: 459.22 LogP: 4.70 TPSA: 96.88
Target Binding affinity: 7.65 GroupD Binding affinity: 5.11 RAscore: 0.98

96) CCNCc1cccnc1NCc1cccnc1NCc1ccncc1-c1ccccc1CF


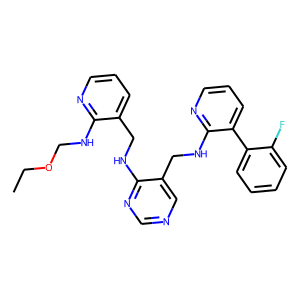


Molecular Weight: 456.24 LogP: 5.34 TPSA: 74.76
Target Binding affinity: 7.70 GroupD Binding affinity: 5.08 RAscore: 0.99

97) COCCCCNc1ncccc1CNc1ncccc1-c1cnccc1NCc1ccco1


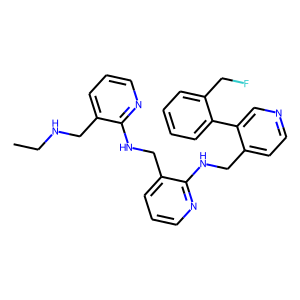


Molecular Weight: 458.24 LogP: 5.19 TPSA: 97.13
Target Binding affinity: 6.97 GroupD Binding affinity: 5.05 RAscore: 0.99

98) CCNCc1cccnc1NCc1cccnc1Nc1ncccc1C(=O)c1ccccc1C


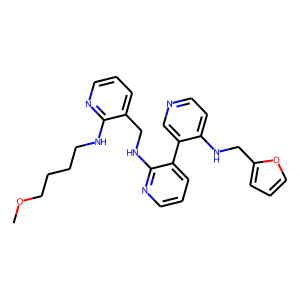


Molecular Weight: 452.23 LogP: 4.88 TPSA: 91.83
Target Binding affinity: 7.42 GroupD Binding affinity: 5.09 RAscore: 0.99

99) CCOCCOc1ccccc1CNc1ncccc1CNc1ncccc1NCc1cc[nH]c1


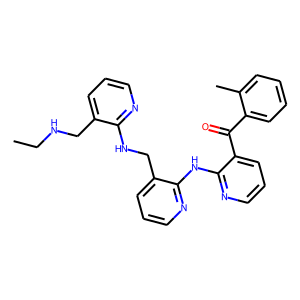


Molecular Weight: 472.26 LogP: 5.06 TPSA: 96.12
Target Binding affinity: 7.16 GroupD Binding affinity: 5.07 RAscore: 0.98

100) CCCCNNc1ncccc1CNc1ncccc1CNc1ncccc1CNC(C)(C)C


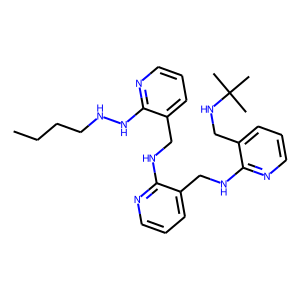


Molecular Weight: 462.32 LogP: 4.70 TPSA: 98.82
Target Binding affinity: 7.42 GroupD Binding affinity: 5.08 RAscore: 0.97
